# Supplementary material for: Chicken Juice Enhances C. jejuni NCTC 11168 Biofilm Formation with Distinct Morphological Features and Altered Protein Expression
Source: Foods. 2024 Jun 11;13(12):1828. doi: 10.3390/foods13121828 (PMC11202532; doi:10.3390/foods13121828)
Supplement: Supplementary file 1 [file foods-13-01828-s001.zip › foods-3050455-supplementary.pdf]

Table. S1. Proteins identified from *C. jejuni* biofilms.

Locus Tag	Genes	Description	COG	Log2(Fold Ratio)
Cj0001	dnaA	Chromosomal replication initiator protein DnaA	L	-4.65
Cj0002	dnaN	DNA polymerase III beta subunit (EC 2.7.7.7)	L	-0.97
Cj0003	gyrB	DNA gyrase subunit B (EC 5.99.1.3)	L	-0.47
Cj0004c		Putative periplasmic protein	-	2.43
Cj0005c		putative molybdenum containing oxidoreductase	S	-1.02
Cj0007	gltB	Glutamate synthase [NADPH] large chain (EC 1.4.1.13)	E	-0.19
Cj0008		hypothetical protein	S	0.54
Cj0009	gltD	Glutamate synthase [NADPH] small chain (EC 1.4.1.13)	C	-3.09
Cj0010c	rnhB	Ribonuclease HII (EC 3.1.26.4)	L	2.14
Cj0011c		hypothetical protein	L	26.72
Cj0012c	rrc	Rubrerythrin	C	-1.19
Cj0013	ilvD	Dihydroxy-acid dehydratase (EC 4.2.1.9)	EG	-1.37
Cj0014c		hypothetical protein	S	24.79
Cj0015c		Hydrolase, HAD superfamily	S	-0.79
Cj0016		7-cyano-7-deazaguanine synthase (EC 6.3.4.20)	F	-7.78
Cj0017c	dsbI	ATP/GTP-binding protein	C	-1.88
Cj0019c		hypothetical protein	NT	-1.77
Cj0020c		Cytochrome c551 peroxidase (EC 1.11.1.5)	C	2.14
Cj0021c		2-keto-4-pentenoate hydratase/2-oxohepta-3-ene-1,7-dioic acid hydratase (catechol pathway)	Q	0.06
Cj0022c		Uncharacterized RNA pseudouridine synthase HP0347	J	-22.40
Cj0023	purB	Adenylosuccinate lyase (EC 4.3.2.2) @ SAICAR lyase (EC 4.3.2.2)	F	-1.22
Cj0024	nrdA	Ribonucleotide reductase of class Ia (aerobic), alpha subunit (EC 1.17.4.1)	F	-0.56
Cj0025c		Putative transmembrane symporter	U	2.33
Cj0026c	thyX	Thymidylate synthase ThyX (EC 2.1.1.148)	F	0.09
Cj0027	pyrG	CTP synthase (EC 6.3.4.2)	F	-0.79
Cj0028	recJ	Single-stranded-DNA-specific exonuclease RecJ	L	-1.20
Cj0029	ansA	L-asparaginase (EC 3.5.1.1)	EJ	-7.57
Cj0030		hypothetical protein	S	3.00
Cj0031		Putative type IIS restriction /modification enzyme, N-terminal half	LV	-0.73
Cj0033		Integral membrane protein	S	-0.33
Cj0034c		Putative periplasmic protein	S	-2.87
Cj0036		hypothetical protein	S	0.05
Cj0037c		Cytochrome c family protein	C	-0.21
Cj0038c		Membrane protein	-	27.19
Cj0039c	typA	GTP-binding protein TypA/BipA	T	-0.05
Cj0040		hypothetical protein	-	-2.12
Cj0041	fliK	Flagellar hook-length control protein FliK	N	-1.32
Cj0042	flgD	Flagellar basal-body rod modification protein FlgD	N	2.24
Cj0043	flgE	Flagellar hook protein FlgE	N	0.83

Cj0044c		hypothetical protein	L	-3.53
Cj0045c		Hemerythrin domain protein	P	-4.42
Cj0053c	mnmA	tRNA-specific 2-thiouridylase MnmA (EC 2.8.1.13)	J	-2.29
Cj0054c		Predicted Rossmann fold nucleotide-binding protein, possible lysine decarboxylase	S	-0.81
Cj0055c		hypothetical protein	-	-2.70
Cj0056c		hypothetical protein	-	0.00
Cj0057		FIG00973752: TolA-like membrane protein	-	0.00
Cj0058		Bacteriocin resistance protein; peptidase C39	S	27.28
Cj0059c	fliY	Flagellar motor switch protein FliN	N	0.54
Cj0060c	fliM	Flagellar motor switch protein FliM	N	-1.85
Cj0061c	fliA	RNA polymerase sigma factor for flagellar operon	K	-2.85
Cj0062c		Motility integral membrane protein	-	-23.54
Cj0063c		Flagellar synthesis regulator FleN	D	-3.43
Cj0064c	flhF	Flagellar biosynthesis protein FlhF	N	0.93
Cj0065c	folK	2-amino-4-hydroxy-6-hydroxymethyldihydropteridine pyrophosphokinase (EC 2.7.6.3)	H	27.25
Cj0066c	aroQ	3-dehydroquinate dehydratase II (EC 4.2.1.10)	E	-1.51
Cj0067		Adenosine deaminase (EC 3.5.4.4), alternative form	F	25.59
Cj0068	pspA	protease IV (PspA)	OU	0.08
Cj0069		hypothetical protein	HJ	-0.79
Cj0073c		Predicted L-lactate dehydrogenase, hypothetical protein subunit YkgG	S	-0.50
Cj0074c		Predicted L-lactate dehydrogenase, Iron-sulfur cluster-binding subunit YkgF	C	-0.21
Cj0075c		Predicted L-lactate dehydrogenase, Fe-S oxidoreductase subunit YkgE	C	-0.51
Cj0076c	lctP	L-lactate permease	C	-0.63
Cj0077c	cdtC	Cytolethal distending toxin subunit C	S	-0.90
Cj0078c	cdtB	Cytolethal distending toxin subunit B, DNase I-like	S	-28.24
Cj0079c	cdtA	Cytolethal distending toxin subunit A	M	-26.86
Cj0081	cydA	Cytochrome d ubiquinol oxidase subunit I (EC 1.10.3.-)	C	-1.55
Cj0085c		Uncharacterized protein YgeA of aspartate/glutamate/hydantoin racemase family	M	-0.14
Cj0086c	ung	Uracil-DNA glycosylase, family 1 (EC 3.2.2.27)	L	2.00
Cj0087	aspA	Aspartate ammonia-lyase (EC 4.3.1.1)	E	-1.39
Cj0088	dcuA	Anaerobic C4-dicarboxylate transporter DcuA	U	-1.22
Cj0089		putative lipoprotein	S	-2.20
Cj0090		putative lipoprotein	S	-24.21
Cj0091		putative lipoprotein	M	-1.93
Cj0092		Putative periplasmic protein	-	0.61
Cj0093		Putative periplasmic protein	M	29.65
Cj0094	rplU	LSU ribosomal protein L21p	J	-1.05
Cj0095	rpmA	LSU ribosomal protein L27p	J	-0.74
Cj0096	obgE	GTP-binding protein Obg	S	-3.14
Cj0097	proB	Glutamate 5-kinase (EC 2.7.2.11)	E	-26.85
Cj0098	fmt	Methionyl-tRNA formyltransferase (EC 2.1.2.9)	J	-3.89

Cj0099	birA	Biotin--protein ligase (EC 6.3.4.9)(EC 6.3.4.10)(EC 6.3.4.11)(EC 6.3.4.15)	H	2.36
Cj0100		Chromosome (plasmid) partitioning protein ParA	D	-1.09
Cj0101		Chromosome (plasmid) partitioning protein ParB	K	0.88
Cj0102	atpF'	ATP synthase F0 sector subunit b' (EC 3.6.3.14)	C	-1.22
Cj0103	atpF	ATP synthase F0 sector subunit b (EC 3.6.3.14)	C	-1.91
Cj0104	atpH	ATP synthase delta chain (EC 3.6.3.14)	C	1.75
Cj0105	atpA	ATP synthase alpha chain (EC 3.6.3.14)	C	-0.34
Cj0106	atpG	ATP synthase gamma chain (EC 3.6.3.14)	C	-1.90
Cj0107	atpD	ATP synthase beta chain (EC 3.6.3.14)	F	-0.65
Cj0108	atpC	ATP synthase epsilon chain (EC 3.6.3.14)	C	-3.93
Cj0109	exbB3	Tol-Pal system protein TolQ	U	0.27
Cj0110	exbD3	Tol biopolymer transport system, TolR protein	U	-0.51
Cj0111		TolA protein	M	1.22
Cj0112	tolB	Tol-Pal system beta propeller repeat protein TolB	U	-1.75
Cj0113	pal	Tol-Pal system peptidoglycan-associated lipoprotein PAL	M	-29.21
Cj0114		Cell division coordinator CpoB	S	0.16
Cj0115	slyD	FKBP-type peptidyl-prolyl cis-trans isomerase SlyD (EC 5.2.1.8)	O	-1.41
Cj0116	fabD	Malonyl CoA-acyl carrier protein transacylase (EC 2.3.1.39)	I	0.90
Cj0117	pfs	Aminodeoxyfutalosine nucleosidase (EC 3.2.2.30)	E	-0.71
Cj0118		tRNA-(cytosine32)-2-thiocytidine synthetase TtcA	H	-27.44
Cj0119		Nicotinamidase (EC 3.5.1.19)	Q	-24.43
Cj0120		hypothetical protein	L	27.54
Cj0121		Metal-dependent hydrolase YbeY, involved in rRNA and/or ribosome maturation and assembly	J	-2.44
Cj0122		hypothetical protein	-	28.67
Cj0123c		tRNA-dihydrouridine synthase DusB	H	23.67
Cj0124c		Probable membrane protein Cj0124c	-	-1.49
Cj0125c		RNA polymerase-binding transcription factor DksA	T	0.96
Cj0126c		23S rRNA (pseudouridine(1915)-N(3))-methyltransferase (EC 2.1.1.177)	J	2.30
Cj0127c	accD	Acetyl-coenzyme A carboxyl transferase beta chain (EC 6.4.1.2)	I	-1.83
Cj0128c		Putative fructose-1,6-bisphosphatase or related enzymes of inositol monophosphatase family	G	-28.40
Cj0129c		Outer membrane protein assembly factor YaeT	M	-0.92
Cj0130	tyrA	Prephenate and/or arogenate dehydrogenase (unknown specificity) (EC 1.3.1.12)(EC 1.3.1.43)	E	0.13
Cj0131		Putative periplasmic protein	M	-1.45
Cj0132	lpxC	UDP-3-O-[3-hydroxymyristoyl] N-acetylglucosamine deacetylase (EC 3.5.1.108)	M	0.96
Cj0133		tRNA threonylcarbamoyladenosine biosynthesis protein TsaB	O	2.67
Cj0134	thrB	Homoserine kinase (EC 2.7.1.39)	F	1.95
Cj0135		Hypothetical protein Cj0135	K	0.00
Cj0136	infB	Translation initiation factor 2	J	-0.01

Cj0137	rbfA	Ribosome-binding factor A	J	1.33
Cj0138		Bacterial ribosome SSU maturation protein RimP	J	-26.97
Cj0139		McrBC restriction endonuclease system, McrB subunit, putative	L	-0.84
Cj0140		hypothetical protein	V	-29.48
Cj0142c		Zinc ABC transporter, ATP-binding protein ZnuC	P	-28.33
Cj0143c		Zinc ABC transporter, substrate-binding protein ZnuA	P	0.41
Cj0144		Methyl-accepting chemotaxis signal transduction protein	NT	-0.39
Cj0145		PhoX, Predicted phosphatase	S	3.14
Cj0146c	trxB	Thioredoxin reductase (EC 1.8.1.9)	C	-2.22
Cj0147c	trxA	Thioredoxin	O	-0.97
Cj0148c		UPF0102 protein YraN	L	0.00
Cj0149c	hom	Homoserine dehydrogenase (EC 1.1.1.3)	E	-2.16
Cj0150c		Aspartate aminotransferase (EC 2.6.1.1)	E	-1.16
Cj0151c		Putative periplasmic protein	-	28.04
Cj0152c		Membrane protein	S	1.35
Cj0153c		23S rRNA (guanosine(2251)-2'-O)-methyltransferase (EC 2.1.1.185)	J	-2.79
Cj0154c		16S rRNA (cytidine(1402)-2'-O)-methyltransferase (EC 2.1.1.198)	H	4.75
Cj0155c	rpmE	LSU ribosomal protein L31p @ LSU ribosomal protein L31p, zinc-dependent	J	3.24
Cj0156c		16S rRNA (uracil(1498)-N(3))-methyltransferase (EC 2.1.1.193)	J	0.00
Cj0157c		Membrane protein	-	-24.64
Cj0158c		Putative haem-binding lipoprotein	C	-5.07
Cj0159c		6-carboxy-5,6,7,8-tetrahydropterin synthase (EC 4.1.2.50)	H	1.65
Cj0160c		7-carboxy-7-deazaguanine synthase (EC 4.3.99.3)	H	-25.63
Cj0161c	moaA	GTP 3',8-cyclase (EC 4.1.99.22)	H	0.55
Cj0162c		Putative periplasmic protein	-	-29.38
Cj0164c	ubiA	Menaquinone via futalosine polyprenyltransferase (MenA homolog)	H	0.32
Cj0166	miaA	tRNA dimethylallyltransferase (EC 2.5.1.75)	J	-2.28
Cj0169	sodB	Superoxide dismutase [Fe] (EC 1.15.1.1)	C	-0.85
Cj0170		hypothetical protein	Q	-27.17
Cj0172c		Carboxynorspermidine synthase (EC 1.5.1.43)	E	-1.06
Cj0173c	cfbpC	Putative iron-uptake ABC transport system ATP-binding protein	E	-28.05
Cj0174c	cfbpB	Ferric iron ABC transporter, permease protein	P	-27.34
Cj0175c	cfbpA	Ferric iron ABC transporter, iron-binding protein	P	0.24
Cj0177		hypothetical protein	S	-28.21
Cj0178		Putative outer membrane siderophore receptor	P	4.89
Cj0179	exbB1	Ferric siderophore transport system, biopolymer transport protein ExbB	U	0.00
Cj0180	exbD1	Ferric siderophore transport system, biopolymer transport protein ExbD	U	24.51
Cj0181	tonB1	putative TonB-dependent receptor	M	3.56

Cj0182		Transmembrane transport protein	I	0.00
Cj0183		Uncharacterized protein HP_1490	S	-2.67
Cj0184c		Putative serine/threonine protein phosphatase (EC 3.1.3.16)	T	-0.52
Cj0186c		Integral membrane protein TerC	P	27.50
Cj0187c	purN	Phosphoribosylglycinamide formyltransferase (EC 2.1.2.2)	F	-1.21
Cj0188c		NAD(P)H-hydrate epimerase (EC 5.1.99.6) / ADP-dependent (S)-NAD(P)H-hydrate dehydratase (EC 4.2.1.136)	H	-2.50
Cj0189c		hypothetical protein	S	-0.12
Cj0190c		AAA+ ATPase superfamily protein YifB/ComM, associated with DNA recombination	O	3.31
Cj0192c	clpP	ATP-dependent Clp protease proteolytic subunit ClpP (EC 3.4.21.92)	O	-27.93
Cj0193c	tig	Cell division trigger factor (EC 5.2.1.8)	D	0.15
Cj0194	folE	GTP cyclohydrolase I (EC 3.5.4.16) type 1	F	-2.24
Cj0195	fliI	Flagellum-specific ATP synthase FliI	NU	1.83
Cj0196c	purF	Amidophosphoribosyltransferase (EC 2.4.2.14)	F	-0.11
Cj0197c	dapB	4-hydroxy-tetrahydrodipicolinate reductase (EC 1.17.1.8)	E	-0.33
Cj0198c		Replication-associated recombination protein RarA	L	-2.27
Cj0199c		Putative periplasmic protein	-	-0.38
Cj0200c		Putative periplasmic protein	-	4.67
Cj0202c		hypothetical protein	S	29.57
Cj0204		Oligopeptide transporter, OPT family	S	-24.27
Cj0206	thrS	Threonyl-tRNA synthetase (EC 6.1.1.3)	J	-0.40
Cj0207	infC	Translation initiation factor 3	J	0.51
Cj0208		DNA modification methylase (Adenine-specific methyltransferase) (EC 2.1.1.72)	L	-29.41
Cj0224	argC	N-acetyl-gamma-glutamyl-phosphate reductase (EC 1.2.1.38)	E	-7.06
Cj0225		Predicted amino-acid acetyltransferase (EC 2.3.1.1) complementing ArgA function in Arginine Biosynthesis pathway	K	-22.30
Cj0226	argB	N-acetylglutamate kinase (EC 2.7.2.8)	F	-25.06
Cj0227	argD	N-acetylornithine aminotransferase (EC 2.6.1.11) @ N-succinyl-L,L-diaminopimelate aminotransferase (EC 2.6.1.17)	E	2.18
Cj0228c	pcm	Protein-L-isoaspartate O-methyltransferase (EC 2.1.1.77)	H	0.22
Cj0229		Protein YrdA	S	-5.59
Cj0230c		Nicotinate phosphoribosyltransferase (EC 6.3.4.21)	H	0.15
Cj0231c	nrdF	Ribonucleotide reductase of class Ia (aerobic), beta subunit (EC 1.17.4.1)	F	1.01
Cj0233c	pyrE	Orotate phosphoribosyltransferase (EC 2.4.2.10)	F	-28.16
Cj0234c	frr	Ribosome recycling factor	J	-1.43
Cj0237	cynT	Carbonic anhydrase, beta class (EC 4.2.1.1)	P	-1.49
Cj0238		Membrane protein	M	-1.59
Cj0239c		Iron-sulfur cluster assembly scaffold protein IscU/NifU-like	CO	-1.13
Cj0240c	iscS	Cysteine desulfurase (EC 2.8.1.7)	E	0.03

Cj0241c		Hemerythrin domain protein	C	-3.18
Cj0243c		hypothetical protein	-	-0.57
Cj0245	rplT	LSU ribosomal protein L20p	J	1.63
Cj0246c		Putative MCP-domain signal transduction protein	NT	-1.08
Cj0248		Predicted signal transduction protein	T	0.97
Cj0249		hypothetical protein	-	-24.98
Cj0250c		L-Proline/Glycine betaine transporter ProP	EGP	0.00
Cj0251c		Highly acidic protein	-	0.00
Cj0252	moaC	Cyclic pyranopterin monophosphate synthase (EC 4.6.1.17)	H	-1.01
Cj0253		Proposed lipoate regulatory protein YbeD	S	-0.19
Cj0254		hypothetical protein	-	-0.08
Cj0255c	exoA	Exodeoxyribonuclease III (EC 3.1.11.2)	L	1.95
Cj0256		N-linked glycan phosphoethanolamine transferase EptC [Campylobacter]	I	0.43
Cj0258		Putative helix-turn-helix motif protein	S	5.14
Cj0259	pyrC	Dihydroorotase (EC 3.5.2.3)	F	-2.10
Cj0260c		hypothetical protein	-	24.28
Cj0261c		Hypothetical protein Cj0261c	J	0.05
Cj0262c		Methyl-accepting chemotaxis signal transduction protein	NT	-1.65
Cj0263		Zinc transporter ZupT	P	0.00
Cj0264c		Biotin sulfoxide reductase (EC 1.-.-.-) / Free methionine-(S)-sulfoxide reductase	C	-0.22
Cj0265c		putative cytochrome C-type haem-binding periplasmic protein	C	-5.77
Cj0268c		Membrane protease family protein HP0248	O	-1.64
Cj0269c	ilvE	Branched-chain amino acid aminotransferase (EC 2.6.1.42)	E	-1.78
Cj0270		2-hydroxymuconate tautomerase-like protein	G	-30.37
Cj0271		Thiol peroxidase, Bcp-type (EC 1.11.1.15)	O	5.28
Cj0272		Epoxyqueuosine reductase (EC 1.17.99.6) QueH / hypothetical domain	C	-0.79
Cj0273	fabZ	3-hydroxyacyl-[acyl-carrier-protein] dehydratase, FabZ form (EC 4.2.1.59)	I	25.14
Cj0274	lpxA	Acyl-[acyl-carrier-protein]--UDP-N-acetylglucosamine O-acyltransferase (EC 2.3.1.129)	M	-27.75
Cj0275	clpX	ATP-dependent Clp protease ATP-binding subunit ClpX	O	0.00
Cj0276	mreB	Rod shape-determining protein MreB	D	1.06
Cj0277	mreC	Rod shape-determining protein MreC	M	6.87
Cj0279	carB	Carbamoyl-phosphate synthase large chain (EC 6.3.5.5)	F	-1.10
Cj0280		Threonylcarbamoyl-AMP synthase (EC 2.7.7.87)	J	-25.54
Cj0281c	tal	Transaldolase (EC 2.2.1.2)	H	-1.08
Cj0282c	serB	Phosphoserine phosphatase (EC 3.1.3.3)	E	0.06
Cj0283c	cheW	Positive regulator of CheA protein activity (CheW)	NT	-1.01
Cj0284c	cheA	Signal transduction histidine kinase CheA	T	-1.40
Cj0285c	cheV	Chemotaxis protein CheV (EC 2.7.3.-)	T	-0.44
Cj0286c		FIG022708: hypothetical protein	S	0.00
Cj0287c	greA	Transcription elongation factor GreA	K	-1.28
Cj0288c	lpxB	Lipid-A-disaccharide synthase (EC 2.4.1.182)	M	1.07

Cj0289c	peb3	Major antigenic peptide PEB3	S	0.49
Cj0293	surE	5'-nucleotidase SurE (EC 3.1.3.5)	S	-0.72
Cj0294		MoeB/thiF family protein	H	-2.49
Cj0295		hypothetical protein	K	-0.43
Cj0296c	panD	Aspartate 1-decarboxylase (EC 4.1.1.11)	H	-28.06
Cj0297c	panC	Pantoate--beta-alanine ligase (EC 6.3.2.1)	H	-2.29
Cj0298c	panB	3-methyl-2-oxobutanoate hydroxymethyltransferase (EC 2.1.2.11)	H	-2.54
Cj0299		Class D beta-lactamase (EC 3.5.2.6) => OXA-61 family	V	5.64
Cj0300c	modC	Molybdenum ABC transporter ATP-binding protein ModC	P	-0.63
Cj0302c		hypothetical protein	H	-3.13
Cj0303c	modA	Molybdenum ABC transporter, substrate-binding protein ModA	P	-1.93
Cj0304c	bioC	Malonyl-[acyl-carrier protein] O-methyltransferase (EC 2.1.1.197)	Q	26.75
Cj0305c		Pimeloyl-[acyl-carrier protein] methyl ester esterase BioG (EC 3.1.1.85)	S	0.07
Cj0306c	bioF	8-amino-7-oxononanoate synthase (EC 2.3.1.47)	E	-30.91
Cj0307	bioA	Adenosylmethionine-8-amino-7-oxononanoate aminotransferase (EC 2.6.1.62)	H	-0.52
Cj0308c	bioD	Dethiobiotin synthase BioD (EC 6.3.3.3)	H	25.51
Cj0311		LSU ribosomal protein L25p	J	-1.25
Cj0312	pth	Peptidyl-tRNA hydrolase (EC 3.1.1.29)	J	-26.58
Cj0313		Lipopolysaccharide export system permease protein LptG	S	-26.99
Cj0314	lysA	Diaminopimelate decarboxylase (EC 4.1.1.20)	E	-0.86
Cj0315		NagD, Predicted sugar phosphatases of the HAD superfamily	G	0.83
Cj0316	pheA	Chorismate mutase I (EC 5.4.99.5) / Prephenate dehydratase (EC 4.2.1.51)	E	-3.33
Cj0317	hisC	Biosynthetic Aromatic amino acid aminotransferase beta (EC 2.6.1.57) @ Histidinol-phosphate aminotransferase (EC 2.6.1.9)	E	-2.19
Cj0318	fliF	Flagellar M-ring protein FliF	N	-0.60
Cj0319	fliG	Flagellar motor switch protein FliG	N	-7.39
Cj0320	fliH	Flagellar assembly protein FliH	N	-0.58
Cj0321	dxs	1-deoxy-D-xylulose 5-phosphate synthase (EC 2.2.1.7)	H	0.62
Cj0322	perR	Peroxide stress regulator; Ferric uptake regulation protein; Fe2+/Zn2+ uptake regulation proteins	K	-0.18
Cj0323		hypothetical protein	S	2.00
Cj0324	ubiE	Demethylmenaquinone methyltransferase (EC 2.1.1.163)	H	32.01
Cj0325	xseA	Exodeoxyribonuclease VII large subunit (EC 3.1.11.6)	L	3.93
Cj0326	serC	Phosphoserine aminotransferase (EC 2.6.1.52)	E	-1.89
Cj0327		RidA/YER057c/UK114 superfamily, group 2, YoaB-like protein	J	-0.36
Cj0328c	fabH	3-oxoacyl-[acyl-carrier-protein] synthase, KASIII (EC 2.3.1.180)	I	-1.43
Cj0329c	plsX	Phosphate:acyl-ACP acyltransferase PlsX (EC 2.3.1.n2)	I	-1.78

Cj0330c	rpmF	LSU ribosomal protein L32p @ LSU ribosomal protein L32p, zinc-independent	J	1.69
Cj0331c		FIG01269488: protein, clustered with ribosomal protein L32p	S	-0.94
Cj0332c	ndk	Nucleoside diphosphate kinase (EC 2.7.4.6)	F	-0.41
Cj0334	ahpC	Alkyl hydroperoxide reductase subunit C-like protein	O	-1.18
Cj0335	flhB	Flagellar biosynthesis protein FlhB	N	-0.13
Cj0336c	motB	Flagellar motor rotation protein MotB	N	10.41
Cj0337c	motA	Flagellar motor rotation protein MotA	N	-29.44
Cj0338c	polA	DNA polymerase I (EC 2.7.7.7)	L	-0.94
Cj0339		Uncharacterized MFS-type transporter	P	0.00
Cj0340		Inosine-uridine preferring nucleoside hydrolase (EC 3.2.2.1)	F	0.70
Cj0342c	uvrA	Excinuclease ABC subunit A	L	-0.86
Cj0343c		Membrane protein	S	-4.51
Cj0345	trpE	Anthranilate synthase, aminase component (EC 4.1.3.27)	EH	-0.39
Cj0346	trpD (trpGD)	Anthranilate synthase, amidotransferase component (EC 4.1.3.27) / Anthranilate phosphoribosyltransferase (EC 2.4.2.18)	F	0.21
Cj0347	trpF	Phosphoribosylanthranilate isomerase (EC 5.3.1.24)	E	-2.46
Cj0348	trpB	Tryptophan synthase beta chain (EC 4.2.1.20)	E	0.72
Cj0349	trpA	Tryptophan synthase alpha chain (EC 4.2.1.20)	E	1.88
Cj0350		Putative uncharacterized protein FIG019238	-	-1.09
Cj0351	fliN	Flagellar motor switch protein FliN	N	23.96
Cj0352		Probable transmembrane protein Cj0352	N	1.68
Cj0353c		Exopolyphosphatase (EC 3.6.1.11)	FP	0.41
Cj0355c		Two-component regulator	K	-0.81
Cj0356c	folB	Dihydroneopterin aldolase (EC 4.1.2.25)	H	-26.94
Cj0357c		Acyl-phosphate:glycerol-3-phosphate O-acyltransferase PlsY (EC 2.3.1.n3)	I	0.37
Cj0358		Cytochrome c551 peroxidase (EC 1.11.1.5)	C	-0.02
Cj0360	glmM	Phosphoglucosamine mutase (EC 5.4.2.10)	G	-0.71
Cj0361	lspA	Lipoprotein signal peptidase (EC 3.4.23.36)	MU	1.37
Cj0362		Protoporphyrinogen IX oxidase, novel form, HemJ (EC 1.3.-.-)	S	0.00
Cj0363c		Oxygen-independent coproporphyrinogen III oxidase, putative	H	-0.49
Cj0364		hypothetical protein	-	-1.96
Cj0365c	cmeC	Multidrug efflux system, outer membrane factor lipoprotein => CmeC	MU	-0.61
Cj0366c	cmeB	Multidrug efflux system, inner membrane proton/drug antiporter (RND type) => CmeB	V	-3.60
Cj0367c	cmeA	Multidrug efflux system, membrane fusion component => CmeA	M	-0.37
Cj0368c	cmeR	Transcriptional repressor of CmeABC operon, CmeR	K	-3.23
Cj0369c		Ferredoxin domain-containing integral membrane protein	C	-1.49
Cj0370	rpsU	SSU ribosomal protein S21p	J	-5.96
Cj0371		Putative flagellar motility protein	-	-1.70

Cj0372		Similarity with glutathionylspermidine synthase (EC 6.3.1.8), group 2	E	-0.83
Cj0373		Putative D-2-hydroxyacid dehydrogenase	C	-1.21
Cj0374		UPF0234 protein Yitk	S	8.87
Cj0375		putative lipoprotein	-	0.00
Cj0376		Putative periplasmic protein	-	-0.49
Cj0377		Probable AAA family ATPase	O	-3.13
Cj0378c		Membrane protein	C	-26.84
Cj0379c		Probable sulfite oxidase	C	-2.91
Cj0380c		hypothetical protein	-	0.66
Cj0381c	pyrF	Orotidine 5'-phosphate decarboxylase (EC 4.1.1.23)	F	0.37
Cj0382c	nusB	Transcription termination protein NusB	K	-27.22
Cj0383c	ribH	6,7-dimethyl-8-ribityllumazine synthase (EC 2.5.1.78)	H	-0.69
Cj0384c	kdsA	2-Keto-3-deoxy-D-manno-octulosonate-8-phosphate synthase (EC 2.5.1.55)	M	-1.02
Cj0386	engA	GTP-binding protein EngA	S	0.72
Cj0387	aroK	Shikimate kinase I (EC 2.7.1.71)	F	-0.59
Cj0388	trpS	Tryptophanyl-tRNA synthetase (EC 6.1.1.2)	J	-2.41
Cj0389	serS	Seryl-tRNA synthetase (EC 6.1.1.11)	J	-0.67
Cj0390		Uncharacterized membrane anchored protein HP_1479	O	1.37
Cj0391c		hypothetical protein	-	-4.17
Cj0392c	pyk	Pyruvate kinase (EC 2.7.1.40)	G	-0.36
Cj0393c	mqo	Malate:quinone oxidoreductase (EC 1.1.5.4)	C	-2.12
Cj0394c		Pantothenate kinase type III, CoaX-like (EC 2.7.1.33)	F	0.82
Cj0396c		putative lipoprotein	-	0.63
Cj0397c		hypothetical protein	-	-0.80
Cj0398	gatC	Aspartyl-tRNA(Asn) amidotransferase subunit C (EC 6.3.5.6) @ Glutamyl-tRNA(Gln) amidotransferase subunit C (EC 6.3.5.7)	J	-2.81
Cj0399		Putative integral membrane protein HP_0181	S	-1.91
Cj0400	fur	Ferric uptake regulation protein FUR	K	0.73
Cj0401	lysS	Lysyl-tRNA synthetase (class II) (EC 6.1.1.6)	J	-0.35
Cj0402	glyA	Serine hydroxymethyltransferase (EC 2.1.2.1)	E	-1.10
Cj0403		hypothetical protein	L	-0.01
Cj0404		Membrane protein	S	-2.62
Cj0405	aroE	Shikimate 5-dehydrogenase I alpha (EC 1.1.1.25)	E	-27.61
Cj0406c		putative lipoprotein	-	-0.08
Cj0407	lgt	Prolipoprotein diacylglyceryl transferase	M	2.48
Cj0408	frdC	Fumarate reductase cytochrome b subunit	C	-1.38
Cj0409	frdA	Fumarate reductase flavoprotein subunit (EC 1.3.5.4)	C	-2.05
Cj0410	frdB	Fumarate reductase iron-sulfur protein (EC 1.3.5.4)	C	-0.46
Cj0411		ATP/GTP-binding protein	S	0.51
Cj0412		ATP/GTP-binding protein	S	-3.78
Cj0414		Gluconate 2-dehydrogenase (EC 1.1.99.3), membrane-bound, gamma subunit	S	-1.66
Cj0415		Gluconate 2-dehydrogenase (EC 1.1.99.3), membrane-bound, flavoprotein	E	-1.50

Cj0417		hypothetical protein	-	0.00
Cj0418c		SrpA-related protein	M	0.64
Cj0419		Diadenosine tetraphosphate (Ap4A) hydrolase and other HIT family hydrolases	FG	-27.13
Cj0420		Putative periplasmic protein	S	-0.39
Cj0422c		hypothetical protein	-	-25.34
Cj0424		hypothetical protein	S	23.18
Cj0426		Bis-ABC ATPase YbiT	S	-3.57
Cj0427		hypothetical protein	-	-0.84
Cj0428		hypothetical protein	-	-0.38
Cj0429c		FIG000605: protein co-occurring with transport systems (COG1739)	S	5.18
Cj0431		Putative periplasmic ATP /GTP-binding protein	NU	1.53
Cj0432c	murD	UDP-N-acetylmuramoyl-L-alanine--D-glutamate ligase (EC 6.3.2.9)	M	-2.37
Cj0434	pgm	2,3-bisphosphoglycerate-independent phosphoglycerate mutase (EC 5.4.2.12)	G	1.09
Cj0435	fabG	3-oxoacyl-[acyl-carrier protein] reductase (EC 1.1.1.100), FadG	IQ	-1.86
Cj0436		hypothetical protein	S	-27.62
Cj0437	sdhA	Succinate dehydrogenase flavoprotein subunit (EC 1.3.5.1)	C	-0.67
Cj0438	sdhB	Succinate dehydrogenase iron-sulfur protein (EC 1.3.5.1)	C	-2.32
Cj0439	sdhC	Heterodisulfide reductase subunit B-like protein @ Putative succinate dehydrogenase subunit	C	-1.87
Cj0440c		Thiaminase II (EC 3.5.99.2) involved in salvage of thiamin pyrimidine moiety, TenA subgroup with Cys in active site	K	-0.66
Cj0441	acpP	Acyl carrier protein	IQ	1.53
Cj0442	fabF	3-oxoacyl-[acyl-carrier-protein] synthase, KASII (EC 2.3.1.179)	I	-1.97
Cj0443	accA	Acetyl-coenzyme A carboxyl transferase alpha chain (EC 6.4.1.2)	I	1.04
Cj0447		Uridine diphosphate glucose pyrophosphatase (EC 3.6.1.45)	L	-27.23
Cj0448c		Methyl-accepting chemotaxis signal transduction protein	NT	-1.11
Cj0449c		Hypothetical protein Cj0449c	S	-2.98
Cj0451	rep	Ribulose-phosphate 3-epimerase (EC 5.1.3.1)	G	-1.10
Cj0452	dnaQ	DEDDh 3'-5' exonuclease domain of the epsilon subunit of DNA polymerase III	L	-25.83
Cj0453	thiC	Phosphomethylpyrimidine synthase ThiC (EC 4.1.99.17)	H	-0.89
Cj0454c		Membrane protein	-	1.17
Cj0455c		putative membrane protein	NU	-30.25
Cj0456c		hypothetical protein	-	-3.23
Cj0457c		putative lipoprotein	S	-1.52
Cj0458c	miaB	tRNA-i(6)A37 methylthiotransferase (EC 2.8.4.3)	J	-0.27
Cj0459c		hypothetical protein	-	-0.49
Cj0460	nusA	Transcription termination protein NusA	K	0.05
Cj0461c		Integral membrane protein	EGP	0.00
Cj0462		Cyclic dehypoxanthine futalosine synthase (EC 1.21.98.1)	H	-0.42

Cj0463		FIG015287: Zinc protease	S	-1.74
Cj0464	recG	ATP-dependent DNA helicase RecG (EC 3.6.4.12)	L	0.27
Cj0465c	ctb	hypothetical protein	S	-1.63
Cj0466	nssR	Transcriptional regulator	K	-0.16
Cj0468		ABC transporter, permease protein (cluster 3, basic aa/glutamine/opines)	P	0.00
Cj0469		ABC transporter, ATP-binding protein (cluster 3, basic aa/glutamine/opines)	E	2.37
Cj0470	tuf	Translation elongation factor Tu	J	1.16
Cj0471	rpmG	LSU ribosomal protein L33p @ LSU ribosomal protein L33p, zinc-independent	J	0.00
Cj0473	nusG	Transcription antitermination protein NusG	K	0.11
Cj0474	rplK	LSU ribosomal protein L11p (L12e)	J	5.06
Cj0475	rplA	LSU ribosomal protein L1p (L10Ae)	J	0.13
Cj0476	rplJ	LSU ribosomal protein L10p (P0)	J	-0.27
Cj0477	rplL	LSU ribosomal protein L7p/L12p (P1/P2)	J	1.56
Cj0478	rpoB	DNA-directed RNA polymerase beta subunit (EC 2.7.7.6)	K	-1.23
Cj0479	rpoC	DNA-directed RNA polymerase beta' subunit (EC 2.7.7.6)	K	-0.77
Cj0480c		Transcriptional regulator, IclR family	K	2.12
Cj0481	dapA	Dihydrodipicolinate synthase family	EM	-0.82
Cj0482	uxaA'	hypothetical protein	G	-27.26
Cj0483	uxaA'	Altronate dehydratase (EC 4.2.1.7)	G	-23.76
Cj0485		L-fuco-beta-pyranose dehydrogenase, type 2 (EC 1.1.1.122)	IQ	-27.74
Cj0487		L-fuconolactone hydrolase	H	-0.48
Cj0488		L-fucose mutarotase (EC 5.1.3.29)	S	-27.96
Cj0490	ald'	Aldehyde dehydrogenase A (EC 1.2.1.22) @ Glycolaldehyde dehydrogenase (EC 1.2.1.21)	C	-2.10
Cj0491	rpsL	SSU ribosomal protein S12p (S23e)	J	0.00
Cj0492	rpsG	SSU ribosomal protein S7p (S5e)	J	-1.39
Cj0493	fusA	Translation elongation factor G	J	0.00
Cj0495		tRNA(1)(Val) (adenine(37)-N(6))-methyltransferase (EC 2.1.1.223)	S	0.00
Cj0496		hypothetical protein	S	2.39
Cj0497		Uncharacterized protein HP0275	S	1.21
Cj0498	trpC	Indole-3-glycerol phosphate synthase (EC 4.1.1.48)	E	0.52
Cj0499		HIT family protein	FG	-3.43
Cj0500		Selenophosphate-dependent tRNA 2-selenouridine synthase	H	-2.40
Cj0503c	hemH	Ferrochelatase, protoheme ferro-lyase (EC 4.99.1.1)	H	2.37
Cj0504c		Predicted dehydrogenases and related proteins	S	2.85
Cj0505c		Putative aminotransferase	E	-2.15
Cj0506	alaS	Alanyl-tRNA synthetase (EC 6.1.1.7)	J	-0.26
Cj0507	maf	Septum formation protein Maf	D	-0.23
Cj0508	pbpA	Multimodular transpeptidase-transglycosylase (EC 2.4.1.129) (EC 3.4.-.-)	M	-1.98
Cj0509c	clpB	Chaperone protein ClpB (ATP-dependent unfoldase)	O	0.81
Cj0510c		hypothetical protein	S	0.56
Cj0511		Putative carboxyl-terminal protease (EC 3.4.21.-)	M	-0.64

Cj0512	purC	Phosphoribosylaminoimidazole-succinocarboxamide synthase (EC 6.3.2.6)	F	-2.08
Cj0513	purS	Phosphoribosylformylglycinamidine synthase, PurS subunit (EC 6.3.5.3)	F	-0.91
Cj0514	purQ	Phosphoribosylformylglycinamidine synthase, glutamine amidotransferase subunit (EC 6.3.5.3)	F	-28.77
Cj0515		Putative periplasmic protein	-	6.66
Cj0516	plsC	Acyl-CoA:1-acyl-sn-glycerol-3-phosphate acyltransferase (EC 2.3.1.51)	I	1.99
Cj0518	htpG	Chaperone protein HtpG	O	-0.55
Cj0519		hypothetical protein	P	26.80
Cj0520		Membrane protein	-	2.11
Cj0524		Sodium-dependent phosphate transporter	P	0.38
Cj0525c	pbpB	Cell division protein FtsI [Peptidoglycan synthetase] (EC 2.4.1.129)	M	-3.49
Cj0526c	fliE	Flagellar hook-basal body complex protein FliE	N	-29.45
Cj0527c	flgC	Flagellar basal-body rod protein FlgC	N	2.07
Cj0528c	flgB	Flagellar basal-body rod protein FlgB	N	-3.39
Cj0529c		Murein endolytic transglycosylase MltG	S	-0.29
Cj0530		Uncharacterized membrane-anchored protein HP0586	M	-0.71
Cj0531	icd	Isocitrate dehydrogenase [NADP] (EC 1.1.1.42); Monomeric isocitrate dehydrogenase [NADP] (EC 1.1.1.42)	C	-0.49
Cj0532	mdh	Malate dehydrogenase (EC 1.1.1.37)	C	0.70
Cj0533	sucC	Succinyl-CoA ligase [ADP-forming] beta chain (EC 6.2.1.5)	F	-1.12
Cj0534	sucD	Succinyl-CoA ligase [ADP-forming] alpha chain (EC 6.2.1.5)	C	-1.22
Cj0535	oorD	2-oxoglutarate/2-oxoacid ferredoxin oxidoreductase, delta subunit, ferredoxin-like 4Fe-4S binding protein (EC 1.2.7.-)	C	-1.14
Cj0536	oorA	2-oxoglutarate/2-oxoacid ferredoxin oxidoreductase, alpha subunit (EC 1.2.7.-)	C	-1.29
Cj0537	oorB	2-oxoglutarate/2-oxoacid ferredoxin oxidoreductase, beta subunit (EC 1.2.7.-)	C	-1.21
Cj0538	oorC	2-oxoglutarate/2-oxoacid ferredoxin oxidoreductase, gamma subunit (EC 1.2.7.-)	C	-0.50
Cj0539		hypothetical protein	S	-2.33
Cj0540		hypothetical protein	-	-27.36
Cj0541		All-trans-hexaprenyl-diphosphate synthase (EC 2.5.1.33)	H	-0.85
Cj0542	hemA	Glutamyl-tRNA reductase (EC 1.2.1.70)	H	-2.06
Cj0543	proS	Prolyl-tRNA synthetase (EC 6.1.1.15), bacterial type	J	-0.63
Cj0545	hemC	Porphobilinogen deaminase (EC 2.5.1.61)	H	-1.19
Cj0546	ubiD	UbiD family decarboxylase associated with menaquinone via futalosine	H	0.41
Cj0547	flaG	Flagellar protein FlaG	N	-2.04
Cj0548	fliD	Flagellar cap protein FliD	N	-1.74
Cj0549	fliS	Flagellar biosynthesis protein FliS	N	-2.94
Cj0550		hypothetical protein	-	-5.02
Cj0551	efp	Translation elongation factor P	J	-0.22
Cj0552		FIG015373: Membrane protein	S	-0.19

Cj0554		hypothetical protein	S	-0.52
Cj0555		Dicarboxylate carrier protein	P	24.24
Cj0556		putative 2-pyrone-4,6-dicarboxylic acid hydrolase	S	2.15
Cj0558c	proA	Gamma-glutamyl phosphate reductase (EC 1.2.1.41)	E	-0.38
Cj0559		Thioredoxin reductase (EC 1.8.1.9)	O	0.56
Cj0562	dnaB	Replicative DNA helicase (DnaB) (EC 3.6.4.12)	L	0.34
Cj0568		hypothetical protein	-	0.00
Cj0569		hypothetical protein	-	0.00
Cj0570		ATP/GTP-binding protein	-	31.23
Cj0571		Transcriptional regulator, YafY family	K	0.13
Cj0572	ribA	3,4-dihydroxy-2-butanone 4-phosphate synthase (EC 4.1.99.12) / GTP cyclohydrolase II (EC 3.5.4.25)	H	-1.10
Cj0573		Transamidase GatB domain protein	S	-0.65
Cj0574	ilvI	Acetolactate synthase large subunit (EC 2.2.1.6)	H	-0.70
Cj0575	ilvH	Acetolactate synthase small subunit (EC 2.2.1.6)	E	0.32
Cj0576	lpxD	UDP-3-O-[3-hydroxymyristoyl] glucosamine N-acyltransferase (EC 2.3.1.191)	M	-1.69
Cj0577c	queA	S-adenosylmethionine:tRNA ribosyltransferase-isomerase (EC 2.4.99.17)	F	2.67
Cj0578c	tatC	Twin-arginine translocation protein TatC	U	-25.42
Cj0579c		Twin-arginine translocation protein TatB	U	-23.97
Cj0580c		Oxygen-independent coproporphyrinogen-III oxidase-like protein YggW	H	0.74
Cj0581		Adenosine (5')-pentaphospho-(5'')-adenosine pyrophosphohydrolase	L	-26.81
Cj0582	lysC	Aspartokinase (EC 2.7.2.4)	E	-0.55
Cj0583		hypothetical protein	S	-26.01
Cj0584		DNA poymerase III subunit delta' (EC 2.7.7.7)	L	28.05
Cj0585	folP	Alternative dihydrofolate reductase 2 / Dihydropteroate synthase (EC 2.5.1.15)	H	0.18
Cj0586	ligA	DNA ligase (NAD(+)) (EC 6.5.1.2)	L	-2.80
Cj0587		Membrane protein	-	26.04
Cj0588	tlyA	RNA binding methyltransferase FtsJ like	J	-24.14
Cj0589	ribF	FMN adenylyltransferase (EC 2.7.7.2) / Riboflavin kinase (EC 2.7.1.26)	H	-23.53
Cj0590		Carboxy-S-adenosyl-L-methionine synthase	J	-27.21
Cj0593c		UPF0126 inner membrane protein YicG	S	0.00
Cj0594c		DNA/RNA non-specific endonuclease	F	-24.24
Cj0595c	nth	Endonuclease III (EC 4.2.99.18)	L	-27.45
Cj0596	peb4/cbf2	Cell binding factor 2 precursor	M	0.04
Cj0597	fba	Fructose-bisphosphate aldolase class II (EC 4.1.2.13)	G	-0.55
Cj0598		Membrane protein	U	-0.08
Cj0599		Putative periplasmic protein	M	-3.81
Cj0600		pyridoxal phosphate-dependent deaminase, putative	E	1.22
Cj0601c		Sodium-dependent transporter, SNF family	P	2.45
Cj0602c		hypothetical protein	S	0.88

Cj0603c	dsbD	Cytochrome c-type biogenesis protein DsbD, protein-disulfide reductase (EC 1.8.1.8)	CO	-32.49
Cj0604		Polyphosphate kinase 2 (EC 2.7.4.1)	S	-1.28
Cj0605		Acetylornithine deacetylase type II (EC 3.5.1.16)	E	-2.96
Cj0606		ABC transporter, RND-adapter-like protein	M	1.14
Cj0607		Macrolide export ATP-binding/permease protein MacB	V	0.83
Cj0608		Efflux transport system, outer membrane factor (OMF) lipoprotein	MU	2.45
Cj0609c		Putative periplasmic protein	E	-4.79
Cj0610c		Putative periplasmic protein	S	-4.62
Cj0611c		Probable poly(beta-D-mannuronate) O-acetylase (EC 2.3.1.-)	M	-24.31
Cj0612c	cft	Bacterial non-heme ferritin (EC 1.16.3.2)	P	-1.49
Cj0613	pstS	Phosphate ABC transporter, substrate-binding protein PstS (TC 3.A.1.7.1)	P	-2.26
Cj0615	pstA	Phosphate ABC transporter, permease protein PstA (TC 3.A.1.7.1)	P	4.66
Cj0616	pstB	Phosphate ABC transporter, ATP-binding protein PstB (TC 3.A.1.7.1)	P	3.36
Cj0618		hypothetical protein	E	23.82
Cj0620		Predicted metal-dependent hydrolase	S	28.94
Cj0621		Protein hydE	O	-0.62
Cj0622	hypF	Acylphosphate phosphohydrolase (EC 3.6.1.7) / [NiFe] hydrogenase metallocenter assembly protein HypF	O	-0.38
Cj0623	hypB	[NiFe] hydrogenase nickel incorporation-associated protein HypB	KO	-2.13
Cj0624	hypC	[NiFe] hydrogenase metallocenter assembly protein HypC	O	-2.90
Cj0625	hypD	[NiFe] hydrogenase metallocenter assembly protein HypD	O	-3.26
Cj0626	hypE	[NiFe] hydrogenase metallocenter assembly protein HypE	O	-0.88
Cj0627	hypA	[NiFe] hydrogenase nickel incorporation protein HypA	S	-3.75
Cj0628		hypothetical protein	S	-6.03
Cj0630c		DNA polymerase III delta subunit (EC 2.7.7.7)	L	-27.94
Cj0631c		3'-to-5' exoribonuclease RNase R	K	1.60
Cj0632	ilvC	Ketol-acid reductoisomerase (NADP(+)) (EC 1.1.1.86)	H	3.23
Cj0633		Putative periplasmic protein	S	25.40
Cj0634	dprA	SMF family protein, DNA processing chain A (DprA)	LU	1.39
Cj0635		Putative pre-16S rRNA nuclease YqgF	J	-28.57
Cj0636		NOL1/NOP2/sun family protein	J	3.12
Cj0637c	mrsA	Peptide-methionine (S)-S-oxide reductase MsrA (EC 1.8.4.11)	O	-2.23
Cj0638c	ppa	Inorganic pyrophosphatase (EC 3.6.1.1)	C	-1.20
Cj0639c	adk	Adenylate kinase (EC 2.7.4.3)	F	-1.53
Cj0640c	aspS	Aspartyl-tRNA synthetase (EC 6.1.1.12) @ Aspartyl-tRNA(Asn) synthetase (EC 6.1.1.23)	J	-4.97
Cj0641	pnk	NAD kinase (EC 2.7.1.23)	F	1.82
Cj0642	recN	DNA repair protein RecN	L	-1.66
Cj0643	cbrR	Two-component system response regulator protein	T	-0.62

Cj0644		Uncharacterized metal-dependent hydrolase YcfH	L	2.08
Cj0645		Membrane-bound lytic murein transglycosylase D	M	-1.97
Cj0646		Septum-associated rare lipoprotein A	M	27.69
Cj0647		3-deoxy-D-manno-octulosonate 8-phosphate phosphatase (EC 3.1.3.45)	M	-4.17
Cj0648		Lipopolysaccharide export system protein LptC	S	1.00
Cj0649		Lipopolysaccharide export system protein LptA	S	-28.88
Cj0650	engB	GTP-binding protein EngB	D	-24.42
Cj0652	pbpC	Peptidoglycan D,D-transpeptidase MrdA (EC 3.4.16.4)	M	-2.81
Cj0653c		Xaa-Pro aminopeptidase (EC 3.4.11.9)	E	-0.59
Cj0659c		Putative periplasmic protein	-	1.40
Cj0660c		Putative transmembrane protein	-	25.02
Cj0661c	era	GTP-binding protein Era	S	-2.98
Cj0662c	hslU	ATP-dependent hsl protease ATP-binding subunit HslU	O	-2.93
Cj0663c	hslV	ATP-dependent protease subunit HslV (EC 3.4.25.2)	O	-1.73
Cj0664c	rplI	LSU ribosomal protein L9p	J	-1.40
Cj0665c	argG	Argininosuccinate synthase (EC 6.3.4.5)	E	-1.34
Cj0667		Ribosome-associated heat shock protein implicated in the recycling of the 50S subunit (S4 paralog)	J	-24.67
Cj0668		tRNA threonylcarbamoyladenosine biosynthesis protein TsaE	S	-0.04
Cj0669		Lipopolysaccharide ABC transporter, ATP-binding protein LptB	S	-25.19
Cj0670	rpoN	RNA polymerase sigma-54 factor RpoN	K	-1.62
Cj0671	dcuB	Anaerobic C4-dicarboxylate transporter DcuB	P	-3.68
Cj0677	kdpB	Potassium-transporting ATPase B chain (EC 3.6.3.12) (TC 3.A.3.7.1)	P	0.74
Cj0679	kdpD	Osmosensitive K+ channel histidine kinase KdpD	T	0.00
Cj0680c	uvrB	Excinuclease ABC subunit B	L	-2.09
Cj0681		hypothetical protein	-	-2.82
Cj0682		hypothetical protein	-	-25.31
Cj0683		Putative periplasmic protein	NU	0.00
Cj0684	priA	Helicase PriA essential for oriC/DnaA-independent DNA replication	L	-3.94
Cj0685c	cipA	Possible sugar transferase	S	29.43
Cj0686	ispG	(E)-4-hydroxy-3-methylbut-2-enyl-diphosphate synthase (flavodoxin) (EC 1.17.7.3)	I	-1.57
Cj0687c	flgH	Flagellar L-ring protein FlgH	N	-25.29
Cj0688	pta	BioD-like N-terminal domain / Phosphate acetyltransferase (EC 2.3.1.8)	C	-0.20
Cj0689	ackA	Acetate kinase (EC 2.7.2.1)	F	-0.76
Cj0690c		Possible restriction /modification enzyme	V	4.69
Cj0691		Membrane protein	-	0.13
Cj0692c		Membrane protein	-	-24.44
Cj0693c	mraW	16S rRNA (cytosine(1402)-N(4))-methyltransferase (EC 2.1.1.199)	J	0.57
Cj0694		Peptidyl-prolyl cis-trans isomerase PpiD (EC 5.2.1.8)	O	0.60

Cj0695	ftsA	Cell division protein FtsA	D	-0.94
Cj0696	ftsZ	Cell division protein FtsZ	D	-3.81
Cj0698	flgG	Flagellar basal-body rod protein FlgG	N	-1.81
Cj0699c	glnA	Glutamine synthetase type I (EC 6.3.1.2)	E	-0.80
Cj0700		hypothetical protein	-	-1.04
Cj0701		Uncharacterized protease HP_0169	O	-0.85
Cj0702	purE	N5-carboxyaminoimidazole ribonucleotide mutase (EC 5.4.99.18)	F	-3.91
Cj0703		hypothetical protein	S	-0.78
Cj0704	glyQ	Glycyl-tRNA synthetase alpha chain (EC 6.1.1.14)	J	-1.08
Cj0705		GTP cyclohydrolase 1 type 2 homolog YbgI	S	0.86
Cj0706		FIG137478: Hypothetical protein	S	-0.78
Cj0707	kdtA	3-deoxy-D-manno-octulosonic acid transferase (EC 2.4.99.12)(EC 2.4.99.13)	M	3.47
Cj0708		Uncharacterized RNA pseudouridine synthase HP0956	J	0.75
Cj0709	ffh	Signal recognition particle protein Ffh	U	-2.15
Cj0710	rpsP	SSU ribosomal protein S16p	J	0.88
Cj0711		KH domain RNA binding protein YlqC	S	1.20
Cj0712	rimM	16S rRNA processing protein RimM	J	3.68
Cj0713	trmD	tRNA (guanine(37)-N(1))-methyltransferase (EC 2.1.1.228)	J	-28.51
Cj0714	rplS	LSU ribosomal protein L19p	J	-2.44
Cj0715		5-hydroxyisourate hydrolase (EC 3.5.2.17)	S	-1.23
Cj0716		2-keto-3-deoxy-D-arabino-heptulosonate-7-phosphate synthase II (EC 2.5.1.54)	E	-1.11
Cj0717		FIG138056: a glutathione-dependent thiol reductase	P	-0.67
Cj0718	dnaE	DNA polymerase III alpha subunit (EC 2.7.7.7)	L	0.60
Cj0719c		Pyridoxal phosphate-containing protein YggS	S	-0.92
Cj0722c		Peptide chain release factor N(5)-glutamine methyltransferase (EC 2.1.1.297)	J	-25.75
Cj0723c		Integral membrane zinc-metalloprotease HP0382, M48 family	O	-2.99
Cj0725c	mogA	Molybdopterin adenylyltransferase (EC 2.7.7.75)	H	0.59
Cj0726c	corA	Magnesium and cobalt transport protein CorA	P	-3.12
Cj0727		ABC transporter, substrate-binding protein (cluster 1, maltose/g3p/polyamine/iron)	P	0.00
Cj0728		Putative periplasmic protein	S	-2.74
Cj0729		hypothetical protein	S	28.93
Cj0732		Ferric iron ABC transporter, ATP-binding protein	P	24.90
Cj0734c	hisJ	ABC transporter, substrate-binding protein (cluster 3, basic aa/glutamine/opines)	ET	0.66
Cj0737		Filamentous haemagglutinin domain protein	U	0.92
Cj0753c	tonB3	putative TonB-dependent receptor	M	-0.38
Cj0755	cfrA	Ferric receptor CfrA	P	1.48
Cj0757	hrcA	Heat-inducible transcription repressor HrcA	K	0.00
Cj0758	grpE	Heat shock protein GrpE	O	-0.90
Cj0759	dnaK	Chaperone protein DnaK	O	-0.83
Cj0760		Membrane protein	S	-0.93

Cj0761		hypothetical protein	S	-28.65
Cj0762c	aspB	Aspartate aminotransferase (EC 2.6.1.1)	E	-1.79
Cj0763c	cysE	Serine acetyltransferase (EC 2.3.1.30)	E	0.25
Cj0764c	speA	Arginine decarboxylase (EC 4.1.1.19)	H	-0.16
Cj0765c	hisS	Histidyl-tRNA synthetase (EC 6.1.1.21)	J	-1.79
Cj0766c	tmk	Thymidylate kinase (EC 2.7.4.9)	F	-5.77
Cj0767c	coaD	Phosphopantetheine adenylyltransferase (EC 2.7.7.3)	H	-3.83
Cj0768c		Flavin prenyltransferase UbiX	H	-26.14
Cj0770c		Methionine ABC transporter substrate-binding protein	M	-31.60
Cj0771c		Methionine ABC transporter substrate-binding protein	M	-0.83
Cj0772c		Methionine ABC transporter substrate-binding protein	P	0.27
Cj0773c		Methionine ABC transporter permease protein	P	-27.38
Cj0774c		Methionine ABC transporter ATP-binding protein	P	-2.19
Cj0775c	valS	Valyl-tRNA synthetase (EC 6.1.1.9)	J	0.39
Cj0776c		Probable periplasmic protein Cj0776c	-	-29.42
Cj0777		ATP-dependent DNA helicase UvrD/PcrA/Rep, epsilon proteobacterial type 1	L	2.87
Cj0778	peb2	Major antigenic peptide PEB2	S	1.47
Cj0779	tpx	Thiol peroxidase, Tpx-type (EC 1.11.1.15)	O	-1.06
Cj0780	napA	Periplasmic nitrate reductase (EC 1.7.99.4)	C	-2.35
Cj0781	napG	Ferredoxin-type protein NapG (periplasmic nitrate reductase)	C	-24.37
Cj0782	napH	Polyferredoxin NapH (periplasmic nitrate reductase)	C	0.00
Cj0783	napB	Nitrate reductase cytochrome c550-type subunit	C	-2.81
Cj0784	napL	Periplasmic nitrate reductase component NapL	S	1.38
Cj0786		Small hydrophobic protein	-	0.00
Cj0787		hypothetical protein	-	-1.79
Cj0788		hypothetical protein	-	-3.63
Cj0790	purU	Formyltetrahydrofolate deformylase (EC 3.5.1.10)	F	1.03
Cj0791c		Cysteine desulfurase (EC 2.8.1.7)	E	1.51
Cj0792		hypothetical protein	S	-28.36
Cj0793	flgS	Flagellar sensory histidine kinase FlgS	T	1.07
Cj0794		hypothetical protein	S	-30.72
Cj0795c	murF	UDP-N-acetylmuramoyl-tripeptide--D-alanyl-D-alanine ligase (EC 6.3.2.10)	M	-1.51
Cj0796c		2-hydroxy-6-oxohepta-2,4-dienoate hydrolase	S	-27.10
Cj0797c		Hypothetical protein Cj0797c	S	26.11
Cj0798c	ddl	D-alanine--D-alanine ligase (EC 6.3.2.4)	F	-1.54
Cj0799c	ruvA	Holliday junction ATP-dependent DNA helicase RuvA (EC 3.6.4.12)	L	-31.35
Cj0800c		hypothetical protein	L	-0.33
Cj0801		Peptidoglycan lipid II flippase MurJ	J	-25.22
Cj0802	cysS	Cysteinyl-tRNA synthetase (EC 6.1.1.16)	J	-4.99
Cj0803	msbA	Efflux ABC transporter, permease/ATP-binding protein	V	-0.05
Cj0804	pyrD	Dihydroorotate dehydrogenase (quinone) (EC 1.3.5.2)	F	-1.10
Cj0805		FIG015547: peptidase, M16 family	S	-1.42
Cj0806	dapA	4-hydroxy-tetrahydrodipicolinate synthase (EC 4.3.3.7)	E	-0.77

Cj0807		Enoyl-[acyl-carrier-protein] reductase (EC 1.3.1.-), 7-alpha-HSDH-like => refractory to triclosan	IQ	-1.01
Cj0808c		Small hydrophobic protein	-	26.05
Cj0809c		MBL-fold metallo-hydrolase superfamily	S	-3.31
Cj0810	nadE	NAD synthetase (EC 6.3.1.5)	F	0.98
Cj0811	lpxK	Tetraacyldisaccharide 4'-kinase (EC 2.7.1.130)	M	-1.68
Cj0812	thrC	Threonine synthase (EC 4.2.3.1)	E	-2.10
Cj0813	kdsB	3-deoxy-manno-octulosonate cytidylyltransferase (EC 2.7.7.38)	M	-1.34
Cj0815		hypothetical protein	S	0.37
Cj0817	glnH	ABC transporter, substrate-binding protein (cluster 3, basic aa/glutamine/opines)	ET	1.85
Cj0818		hypothetical protein	-	0.00
Cj0820c	fliP	Flagellar biosynthesis protein FliP	N	0.00
Cj0821	glmU	N-acetylglucosamine-1-phosphate uridyltransferase (EC 2.7.7.23) / Glucosamine-1-phosphate N-acetyltransferase (EC 2.3.1.157)	M	-0.88
Cj0822	dfp	Phosphopantothenoylcysteine decarboxylase (EC 4.1.1.36) / Phosphopantothenoylcysteine synthetase (EC 6.3.2.5)	H	-0.80
Cj0823		hypothetical protein	-	0.00
Cj0824	uppS	Undecaprenyl diphosphate synthase (EC 2.5.1.31)	H	-26.65
Cj0826		Lipopolysaccharide export system permease protein LptF	S	-24.32
Cj0827	truA	tRNA pseudouridine(38-40) synthase (EC 5.4.99.12)	J	2.40
Cj0828c	ilvA	Threonine dehydratase, catabolic (EC 4.3.1.19) @ L-serine dehydratase, (PLP)-dependent (EC 4.3.1.17)	E	-2.31
Cj0829c		CoA-binding domain protein	Q	27.19
Cj0830		Uncharacterized protein jhp1395	S	22.35
Cj0831c	trmA	tRNA (uracil(54)-C5)-methyltransferase (EC 2.1.1.35) @ tmRNA (uracil(341)-C5)-methyltransferase	J	-3.48
Cj0832c		NhaC, Na+/H+ antiporter	C	-24.71
Cj0833c		Oxidoreductase	S	0.19
Cj0834c		Ankyrin repeat-containing possible periplasmic protein	S	1.87
Cj0835c	acnB	Aconitate hydratase 2 (EC 4.2.1.3)	C	-0.86
Cj0836	ogt	Methylated-DNA--protein-cysteine methyltransferase (EC 2.1.1.63)	H	26.84
Cj0837c		hypothetical protein	M	0.00
Cj0838c	metG	Methionyl-tRNA synthetase (EC 6.1.1.10)	J	-0.22
Cj0839c		Uncharacterized protein HP1384	-	-2.55
Cj0840c	fbp	Fructose-1,6-bisphosphatase, type I (EC 3.1.3.11)	G	-1.66
Cj0841c	mobB	Molybdopterin-guanine dinucleotide biosynthesis protein MobB	H	0.00
Cj0842		putative lipoprotein	-	-1.05
Cj0843c		Soluble lytic murein transglycosylase (EC 4.2.2.n1)	M	0.63
Cj0845c	gltX	Glutamyl-tRNA(Gln) synthetase (EC 6.1.1.24)	J	0.76
Cj0846		Predicted phosphohydrolase	S	0.51
Cj0847	psd	Phosphatidylserine decarboxylase (EC 4.1.1.65)	I	-1.58

Cj0848c		Uncharacterized homolog of the cytoplasmic domain of flagellar protein FhlB	S	1.65
Cj0849c		hypothetical protein	-	-5.23
Cj0851c		Probable integral membrane protein Cj0851c	-	-23.90
Cj0854c		Putative periplasmic protein	C	0.00
Cj0855	folD	Methenyltetrahydrofolate cyclohydrolase (EC 3.5.4.9) / Methylenetetrahydrofolate dehydrogenase (NADP+) (EC 1.5.1.5)	F	3.10
Cj0856	lepP	Signal peptidase I (EC 3.4.21.89)	U	-1.69
Cj0857c	moeA	Molybdopterin molybdenumtransferase (EC 2.10.1.1)	H	-0.56
Cj0858c	murA	UDP-N-acetylglucosamine 1-carboxyvinyltransferase (EC 2.5.1.7)	M	-3.16
Cj0861c	pabA	Para-aminobenzoate synthase, amidotransferase component (EC 2.6.1.85)	EH	0.42
Cj0862c	pabB	Para-aminobenzoate synthase, aminase component (EC 2.6.1.85) / Aminodeoxychorismate lyase (EC 4.1.3.38)	EH	-3.96
Cj0863c	xerD	Integrase-recombinase protein XERCD family	L	2.61
Cj0864		Periplasmic thiol:disulfide interchange protein, DsbA-like	O	26.35
Cj0872	dsbA	Periplasmic thiol:disulfide interchange protein, DsbA-like	O	1.51
Cj0874c		Cytochrome c family protein	C	-28.54
Cj0876c		Cytochrome c family protein	C	0.00
Cj0879c		Putative periplasmic protein	-	-30.99
Cj0880c		hypothetical protein	-	-1.95
Cj0881c		3'-to-5' oligoribonuclease B, Bacillus type	S	-0.85
Cj0882c	flhA	Flagellar biosynthesis protein FlhA	N	-3.48
Cj0883c		Rrf2 family transcriptional regulator	K	-1.03
Cj0884	rpsO	SSU ribosomal protein S15p (S13e)	J	0.18
Cj0886c	ftsK	DNA translocase FtsK	D	-1.10
Cj0887c	flgL	Flagellar hook-associated protein FlgL	N	-0.29
Cj0888c		Bis-ABC ATPase Uup	S	1.39
Cj0889c		Two-component system sensor histidine kinase	T	29.13
Cj0890c		Two-component transcriptional response regulator, LuxR family	T	2.09
Cj0891c	serA	D-3-phosphoglycerate dehydrogenase (EC 1.1.1.95)	E	-0.86
Cj0892c		Putative periplasmic protein	-	26.10
Cj0893c	rpsA	SSU ribosomal protein S1p	J	0.29
Cj0894c	ispH	4-hydroxy-3-methylbut-2-enyl diphosphate reductase (EC 1.17.7.4)	IM	0.58
Cj0895c	aroA	3-phosphoshikimate 1-carboxyvinyltransferase (EC 2.5.1.19)	E	-0.43
Cj0898		HIT family protein	FG	-0.17
Cj0899c	thiJ	DJ-1/YajL/PfpI superfamily, includes chaperone protein YajL (former ThiJ), parkinsonism-associated protein DJ-1, peptidases PfpI, Hsp31	S	-0.62
Cj0901		ABC transporter, permease protein (cluster 3, basic aa/glutamine/opines)	P	-27.38
Cj0902	glnQ	ABC transporter, ATP-binding protein (cluster 3, basic aa/glutamine/opines)	E	-4.32

Cj0903c		Putative amino-acid transport protein	E	-1.45
Cj0904c		tRNA (cytidine(34)-2'-O)-methyltransferase (EC 2.1.1.207)	J	-25.22
Cj0905c	alr	Alanine racemase (EC 5.1.1.1)	E	-0.08
Cj0906c		Putative periplasmic protein	S	-0.73
Cj0908		Putative periplasmic protein	-	-24.87
Cj0909		Copper metallochaperone PCu(A)C, inserts Cu(I) into cytochrome oxidase subunit II	S	0.45
Cj0910		Putative periplasmic protein	-	-26.12
Cj0911		Cytochrome oxidase biogenesis protein Sco1/SenC/PrrC, thiol-disulfide reductase involved in Cu(I) insertion into CoxII Cu(A) center	S	-27.44
Cj0912c	cysM	Cysteine synthase (EC 2.5.1.47)	E	0.77
Cj0913c	hupB	DNA-binding protein HU	L	-0.30
Cj0914c	ciaB	Campylobacter invasion antigen B (CiaB)	G	-1.51
Cj0915		Putative acyl-CoA thioester hydrolase (EC 3.1.2.-)	I	-29.91
Cj0916c		Uncharacterized proteins YbdD and YjiX	S	0.75
Cj0917c	cstA	Carbon starvation protein A	T	0.00
Cj0918c	prsA	Ribose-phosphate pyrophosphokinase (EC 2.7.6.1)	F	-1.64
Cj0919c		ABC transporter, permease protein PebF (cluster 3, basic aa/glutamine/opines)	P	-26.58
Cj0920c		ABC transporter, permease protein PebE (cluster 3, basic aa/glutamine/opines)	P	-4.18
Cj0921c	peb1A	ABC transporter, substrate-binding protein PebA (cluster 3, basic aa/glutamine/opines)	ET	0.03
Cj0922c	pebC	ABC transporter, ATP-binding protein PebC (cluster 3, basic aa/glutamine/opines)	E	-3.08
Cj0923c	cheR	Chemotaxis protein methyltransferase CheR (EC 2.1.1.80)	NT	-1.32
Cj0924c	cheB'	Chemotaxis response regulator protein-glutamate methylesterase CheB (EC 3.1.1.61)	NT	-1.84
Cj0925	rpiB	Ribose-5-phosphate isomerase B (EC 5.3.1.6)	G	-24.12
Cj0926		Membrane protein	-	-25.34
Cj0927	apt	Adenine phosphoribosyltransferase (EC 2.4.2.7)	F	-3.27
Cj0928		Membrane protein	S	-23.61
Cj0929	pepA	Cytosol aminopeptidase PepA (EC 3.4.11.1)	E	-3.00
Cj0930		GTP-binding and nucleic acid-binding protein YchF	J	-1.35
Cj0931c	argH	Argininosuccinate lyase (EC 4.3.2.1)	E	-1.39
Cj0932c	pckA	Phosphoenolpyruvate carboxykinase [ATP] (EC 4.1.1.49)	H	-1.06
Cj0933c	pycB	Oxaloacetate decarboxylase Na(+) pump, alpha chain (EC 4.1.1.3)	C	-0.99
Cj0934c		Putative transmembrane transport protein	P	23.88
Cj0935c		Sodium-dependent transporter, SNF family	P	7.13
Cj0937		Auxin efflux carrier family protein	S	-27.07
Cj0938c	aas	Lysophospholipid transporter LplT / 2-acylglycerophosphoethanolamine acyltransferase (EC 2.3.1.40) / Acyl-[acyl-carrier-protein] synthetase (EC 6.2.1.20)	I	0.97
Cj0939c		hypothetical protein	-	-2.17

Cj0941c		Lipoprotein releasing system transmembrane protein LolC/LolE	M	2.70
Cj0942c	secA	Protein translocase subunit SecA	U	-0.65
Cj0943	lolA	Outer membrane lipoprotein carrier protein LolA	M	2.08
Cj0944c		Putative periplasmic protein	-	-8.06
Cj0945c		Putative helicase	L	-25.95
Cj0946		putative lipoprotein	M	-1.42
Cj0947c		N-carbamoylputrescine amidase (EC 3.5.1.53) @ Aliphatic amidase AmiE (EC 3.5.1.4)	S	-4.72
Cj0948c		Ferrous-iron efflux pump FieF	P	0.68
Cj0949c		Agmatine deiminase (EC 3.5.3.12)	E	1.63
Cj0950c		putative lipoprotein	O	-0.32
Cj0951c		methyl-accepting chemotaxis protein (tlpB), putative	NT	2.11
Cj0952c		methyl-accepting chemotaxis protein (tlpB), putative	NT	23.39
Cj0953c	purH	IMP cyclohydrolase (EC 3.5.4.10) / Phosphoribosylaminoimidazolecarboxamide formyltransferase (EC 2.1.2.3)	F	-0.35
Cj0954c		DnaJ-like protein DjlA	O	-1.64
Cj0956c	trmE	tRNA-5-carboxymethylaminomethyl-2-thiouridine(34) synthesis protein MnmE	J	-1.01
Cj0957c		RNA-binding protein Jag	S	-26.00
Cj0958c		Inner membrane protein translocase and chaperone YidC, long form	U	-0.40
Cj0960c	rnpA	Ribonuclease P protein component (EC 3.1.26.5)	J	-1.15
Cj0961c	rpmH	LSU ribosomal protein L34p	J	23.22
Cj0962		Acetyltransferase, GNAT family	K	-2.52
Cj0963		DNA polymerase, bacteriophage-type (EC 2.7.7.7)	L	0.00
Cj0964		Uncharacterized periplasmic protein HP0781	E	-0.31
Cj0965c		4-hydroxybenzoyl-CoA thioesterase family active site	S	-27.21
Cj0967		Putative periplasmic protein	-	25.57
Cj0975		hypothetical protein	U	3.00
Cj0976		tRNA ho5U(34) carboxymethyltransferase	J	-4.30
Cj0978c		putative lipoprotein	-	-23.21
Cj0979c		Thermonuclease family protein	L	-25.52
Cj0980		Cytosol nonspecific dipeptidase (EC 3.4.13.18)	E	-1.81
Cj0981c	cjaB	Transmembrane transport protein	EGP	26.39
Cj0982c	cjaA	ABC transporter, substrate-binding protein (cluster 3, basic aa/glutamine/opines)	ET	0.08
Cj0983		Surface-exposed lipoprotein JlpA	M	0.98
Cj0984		UPF0246 protein YaaA	S	29.33
Cj0985c	hipO	Hippurate hydrolase (EC 3.5.1.32)	S	-2.26
Cj0987c		Integral membrane protein	P	-26.51
Cj0990c		hypothetical protein	S	-24.58
Cj0991c		Putative oxidoreductase ferredoxin-type protein, clusters with CPO	C	0.79
Cj0992c	hemN	Coproporphyrinogen III oxidase, oxygen-independent (EC 1.3.99.22)	H	-1.02

Cj0993c		hypothetical protein	S	-5.39
Cj0994c	argF	Ornithine carbamoyltransferase (EC 2.1.3.3)	E	-0.95
Cj0995c	hemB	Porphobilinogen synthase (EC 4.2.1.24)	H	-1.00
Cj0996	ribA	GTP cyclohydrolase II (EC 3.5.4.25)	F	-23.69
Cj0997		16S rRNA (guanine(527)-N(7))-methyltransferase (EC 2.1.1.170)	J	-2.80
Cj0998c		Putative periplasmic protein	-	-0.42
Cj0999c		UPF0324 inner membrane protein YeiH	S	-25.15
Cj1000		Transcriptional regulator, LysR family	K	0.00
Cj1001	rpoD	RNA polymerase sigma factor RpoD	K	1.04
Cj1002c		phosphohistidine phosphatase SixA, putative	T	-25.97
Cj1005c		ATP-dependent zinc metalloprotease HP0286	O	-0.22
Cj1006c		tRNA t(6)A37-methylthiotransferase (EC 2.8.4.5)	J	4.53
Cj1007c		Uncharacterized integral membrane protein HP0284	M	-0.80
Cj1008c	aroB	3-dehydroquinate synthase (EC 4.2.3.4)	E	-2.07
Cj1009c		TrkA domain protein HP0282	S	1.74
Cj1010	tgt	Queuine tRNA-ribosyltransferase (EC 2.4.2.29)	F	-7.53
Cj1011		Magnesium and cobalt transport protein CorA	P	-24.08
Cj1012c		Membrane protein	-	1.09
Cj1013c		Putative cytochrome C-type biogenesis protein	O	-1.38
Cj1014c	livF	Branched-chain amino acid ABC transporter, ATP-binding protein LivF (TC 3.A.1.4.1)	E	-2.88
Cj1015c	livG	Branched-chain amino acid ABC transporter, ATP-binding protein LivG (TC 3.A.1.4.1)	E	-0.76
Cj1018c	livK	Leucine-specific ABC transporter, substrate-binding protein LivK (TC 3.A.1.4.1)	E	-28.81
Cj1019c	livJ	Branched-chain amino acid ABC transporter, substrate-binding protein LivJ (TC 3.A.1.4.1)	E	1.80
Cj1020c		Putative cytochrome c	C	-2.59
Cj1021c		Putative periplasmic protein	S	0.00
Cj1022c		Membrane protein	S	27.06
Cj1023c	asd	Aspartate-semialdehyde dehydrogenase (EC 1.2.1.11)	E	-0.79
Cj1024c	flgR	Signal-transduction regulatory protein FlgR	T	-2.38
Cj1026c		Flagellar protein FlgP	S	0.63
Cj1027c	gyrA	DNA gyrase subunit A (EC 5.99.1.3)	L	-0.19
Cj1028c		Uncharacterized protein HP_1473	S	24.37
Cj1029c	mapA	Outer membrane liproprotein mapA precursor	-	-0.03
Cj1030c	lepA	Translation elongation factor LepA	M	-0.40
Cj1031	cmeD	Multidrug efflux system, outer membrane factor lipoprotein => CmeD	MU	0.00
Cj1032	cmeE	Multidrug efflux system, membrane fusion component => CmeE	M	0.05
Cj1033	cmeF	Multidrug efflux system, inner membrane proton/drug antiporter (RND type) => CmeF	V	1.18
Cj1034c		Possible dnaJ-like protein	O	0.00
Cj1035c		Arginyl-tRNA--protein transferase (EC 2.3.2.8)	O	1.31
Cj1036c		hypothetical protein	-	2.96

Cj1037c	pycA	Pyruvate carboxylase subunit A (EC 6.4.1.1)	I	-1.00
Cj1039	murG	UDP-N-acetylglucosamine--N-acetylmuramyl-(pentapeptide) pyrophosphoryl-undecaprenol N-acetylglucosamine transferase (EC 2.4.1.227)	M	-28.42
Cj1041c		Putative periplasmic ATP/GTP-binding protein	S	-1.24
Cj1042c		Transcriptional regulator, AraC family	K	1.51
Cj1043c		Thiazole tautomerase TenI (EC 5.3.99.10)	H	-3.70
Cj1044c	thiH	2-iminoacetate synthase (ThiH) (EC 4.1.99.19)	C	1.44
Cj1045c	thiG	Thiazole synthase (EC 2.8.1.10)	H	-1.56
Cj1046c	moeB	Sulfur carrier protein ThiS / Sulfur carrier protein ThiS adenylyltransferase (EC 2.7.7.73)	H	-29.20
Cj1047c	thiS	Sulfur carrier protein ThiS	H	-0.91
Cj1048c	dapE	N-succinyl-L,L-diaminopimelate desuccinylase (EC 3.5.1.18)	E	-0.36
Cj1049c		Membrane protein	E	27.51
Cj1050c	npdA	NAD-dependent protein deacetylase of SIR2 family	K	-6.04
Cj1051c	cjeI	Type I restriction-modification system, DNA-methyltransferase subunit M (EC 2.1.1.72) / Type I restriction-modification system, specificity subunit S	V	1.44
Cj1052c	mutS	Recombination inhibitory protein MutS2	L	-0.17
Cj1053c		Membrane protein	-	29.13
Cj1054c	murC	UDP-N-acetylmuramate--L-alanine ligase (EC 6.3.2.8)	M	-3.94
Cj1055c		Phosphoglycerol transferase-like protein	M	-4.91
Cj1056c		Uncharacterized amidohydrolase HP_1481	S	0.97
Cj1057c		Exodeoxyribonuclease VII small subunit (EC 3.1.11.6)	L	0.00
Cj1058c	guaB	Inosine-5'-monophosphate dehydrogenase (EC 1.1.1.205) / CBS domain	F	-1.25
Cj1059c	gatA	Aspartyl-tRNA(Asn) amidotransferase subunit A (EC 6.3.5.6) @ Glutamyl-tRNA(Gln) amidotransferase subunit A (EC 6.3.5.7)	J	-2.11
Cj1061c	ileS	Isoleucyl-tRNA synthetase (EC 6.1.1.5)	J	-2.22
Cj1062		ADP-ribose pyrophosphatase of COG1058 family (EC 3.6.1.13) / Nicotinamide-nucleotide amidase (EC 3.5.1.42)	S	-1.36
Cj1063		Acetyltransferase	K	-30.65
Cj1066	rdxA	Oxygen-insensitive NAD(P)H nitroreductase (EC 1.-.-.-) / Dihydropteridine reductase (EC 1.5.1.34)	C	-1.45
Cj1069		hypothetical protein	S	-25.15
Cj1070	rpsF	SSU ribosomal protein S6p	J	0.00
Cj1071	ssb	Single-stranded DNA-binding protein	L	-0.43
Cj1072	rpsR	SSU ribosomal protein S18p @ SSU ribosomal protein S18p, zinc-independent	J	-30.10
Cj1073c	lon	ATP-dependent protease La (EC 3.4.21.53) Type I	O	-1.80
Cj1074c		putative lipoprotein	M	-1.34
Cj1075		Flagellar assembly factor FliW	N	-1.26
Cj1076	proC	Pyrroline-5-carboxylate reductase (EC 1.5.1.2)	E	3.16
Cj1078		Putative periplasmic protein	-	0.00
Cj1080c	hemD	Uroporphyrinogen-III synthase (EC 4.2.1.75)	H	27.75
Cj1081c	thiE	Thiamin-phosphate pyrophosphorylase (EC 2.5.1.3)	H	-0.12

Cj1082c	thiD	Hydroxymethylpyrimidine kinase (EC 2.7.1.49) @ Hydroxymethylpyrimidine phosphate kinase ThiD (EC 2.7.4.7)	H	-2.28
Cj1083c		Endonuclease III (EC 4.2.99.18)	L	-28.01
Cj1084c		ATP/GTP-binding protein	S	-5.52
Cj1085c	mfd	Transcription-repair coupling factor	L	0.96
Cj1086c		Polymer-forming bactofilin	M	0.00
Cj1087c		Peptidase, M23/M37 family	M	5.64
Cj1088c	folC	Dihydrofolate synthase (EC 6.3.2.12) @ Folylpolyglutamate synthase (EC 6.3.2.17)	H	2.15
Cj1089c		hypothetical protein	T	-4.03
Cj1090c		Probable lipoprotein Cj1090c	S	-25.49
Cj1092c	secF	Protein translocase subunit SecF	U	-28.65
Cj1093c	secD	Protein translocase subunit SecD	U	-1.51
Cj1094c	yajC	Protein translocase subunit YajC	U	-27.29
Cj1095		Apolipoprotein N-acyltransferase / Copper homeostasis protein CutE	M	-24.38
Cj1096c	metK	S-adenosylmethionine synthetase (EC 2.5.1.6)	H	-1.75
Cj1097		Serine/threonine:Na+ symporter SstT	E	-26.36
Cj1098	pyrB	Aspartate carbamoyltransferase (EC 2.1.3.2)	F	-32.94
Cj1099		Oligoendopeptidase F-like protein	E	-1.26
Cj1100		hypothetical protein	-	27.01
Cj1101		ATP-dependent DNA helicase UvrD/PcrA/Rep, epsilon proteobacterial type 2	L	-4.74
Cj1102	truB	tRNA pseudouridine(55) synthase (EC 5.4.99.25)	J	-2.58
Cj1103	csrA	Carbon storage regulator	J	0.63
Cj1104		4-diphosphocytidyl-2-C-methyl-D-erythritol kinase (EC 2.7.1.148)	F	-29.95
Cj1105	smpB	tmRNA-binding protein SmpB	J	-24.71
Cj1106		Possible periplasmic thioredoxin	CO	-27.61
Cj1107	clpS	ATP-dependent Clp protease adaptor protein ClpS	S	34.77
Cj1108	clpA	ATP-dependent Clp protease ATP-binding subunit ClpA	O	-2.27
Cj1109	aat	Leucyl/phenylalanyl-tRNA--protein transferase (EC 2.3.2.6)	O	25.56
Cj1110c		Methyl-accepting chemotaxis sensor/transducer protein	NT	-1.26
Cj1111c		MarC family integral membrane protein	U	-26.85
Cj1112c		Peptide-methionine (R)-S-oxide reductase MsrB (EC 1.8.4.12)	C	-2.80
Cj1113		FIG00005326: uncharacterized protein	S	-0.30
Cj1114c	pssA	CDP-diacylglycerol--serine O-phosphatidyltransferase (EC 2.7.8.8)	I	0.00
Cj1115c		Phosphatidylserine decarboxylase-related protein	I	-27.72
Cj1116c	ftsH	Cell division-associated, ATP-dependent zinc metalloprotease FtsH	O	-1.08
Cj1117c	prmA	Ribosomal protein L11 methyltransferase	J	-2.13
Cj1118c	cheY	Chemotaxis regulator - transmits chemoreceptor signals to flagellar motor components CheY	KT	0.16
Cj1119c	pglG	N-linked glycosylation glycosyltransferase PglG	NT	-0.23

Cj1120c	pglF	UDP-N-acetylglucosamine 4,6-dehydratase (EC 4.2.1.135)	GM	-0.53
Cj1121c	pglE	UDP-N-acetylbacillosamine transaminase (EC 2.6.1.34)	E	-2.45
Cj1124c	pglC	Undecaprenyl phosphate N,N'-diacetylbacillosamine 1-phosphate transferase (EC 2.7.8.36)	M	2.71
Cj1125c	pglA	N,N'-diacetylbacillosaminyl-diphospho-undecaprenol alpha-1,3-N-acetylgalactosaminyltransferase (ED 2.4.1.290)	M	-1.39
Cj1126c	pglB	Oligosaccharyltransferase PglB (EC 2.4.1.119)	S	-31.08
Cj1127c	pglJ	N-acetylgalactosamine-N,N'-diacetylbacillosaminyl-diphospho-undecaprenol 4-alpha-N-acetylgalactosaminyltransferase (EC 2.4.1.291)	M	-0.52
Cj1128c	pglI	GalNAc(5)-diNAcBac-PP-undecaprenol beta-1,3-glucosyltransferase (EC 2.4.1.293)	M	-1.98
Cj1129c	pglH	GalNAc-alpha-(1->4)-GalNAc-alpha-(1->3)-diNAcBac-PP-undecaprenol alpha- 1,4-N-acetyl-D-galactosaminyltransferase (EC 2.4.1.292)	M	-3.48
Cj1130c	pglK	Multi-drug resistance efflux ABC transporter, permease/ATP-binding protein HetA	P	0.25
Cj1131c	gne	UDP-glucose 4-epimerase (EC 5.1.3.2)	M	-1.85
Cj1132c		Polysaccharide biosynthesis protein WlaX	L	1.41
Cj1133	waaC	Lipopolysaccharide core heptosyltransferase I	M	-1.94
Cj1134	htrB	Lipid A biosynthesis lauroyl acyltransferase (EC 2.3.1.241)	M	2.41
Cj1135		Putative two-domain glycosyltransferase	M	0.42
Cj1136		Beta-1,3-galactosyltransferase / Beta-1,4-galactosyltransferase	M	-29.66
Cj1137c		hypothetical protein	M	22.54
Cj1138		Beta-1,3-galactosyltransferase / Beta-1,4-galactosyltransferase	M	2.66
Cj1139c	wlaN	Beta-1,3-galactosyltransferase / Beta-1,4-galactosyltransferase	S	29.18
Cj1140	cstIII	CMP-N-acetylneuraminate-beta-galactosamide-alpha-2,3-sialyltransferase (EC 2.4.99.-)	G	25.08
Cj1141	neuB1	N-acetylneuraminate synthase (EC 2.5.1.56)	M	-1.85
Cj1142	neuC1	UDP-N-acetylglucosamine 2-epimerase (hydrolyzing) (EC 3.2.1.183)	M	-2.85
Cj1143	neuA1, cgtA	Beta-1,4-N-acetylgalactosaminyltransferase (EC 2.4.1.-)	M	0.34
Cj1146c	waaV	Beta-1,3-galactosyltransferase / Beta-1,4-galactosyltransferase	S	24.23
Cj1148	waaF	ADP-heptose--lipooligosaccharide heptosyltransferase II	M	-27.57
Cj1149c	gmhA	D-sedoheptulose 7-phosphate isomerase (EC 5.3.1.28)	G	-0.02
Cj1150c	hldE	D-glycero-beta-D-manno-heptose 1-phosphate adenylyltransferase (EC 2.7.7.70) / D-glycero-beta-D-manno-heptose-7-phosphate kinase (EC 2.7.1.167)	F	-0.68
Cj1151c	hldD	ADP-L-glycero-D-manno-heptose-6-epimerase (EC 5.1.3.20)	M	-1.73
Cj1152c	gmhB	D-glycero-beta-D-manno-heptose-1,7-bisphosphate 7-phosphatase (EC 3.1.3.82)	E	-24.54
Cj1153		Cytochrome C553 (soluble cytochrome f)	C	-4.73

Cj1154c		Type cbb3 cytochrome oxidase biogenesis protein CcoS, involved in heme b insertion	P	27.87
Cj1155c		Lead, cadmium, zinc and mercury transporting ATPase (EC 3.6.3.3) (EC 3.6.3.5); Copper-translocating P-type ATPase (EC 3.6.3.4)	P	1.00
Cj1156	rho	Transcription termination factor Rho	K	-1.26
Cj1157	dnaX	DNA polymerase III subunits gamma and tau (EC 2.7.7.7)	H	-2.77
Cj1161c		Lead, cadmium, zinc and mercury transporting ATPase (EC 3.6.3.3) (EC 3.6.3.5); Copper-translocating P-type ATPase (EC 3.6.3.4)	P	-3.87
Cj1162c		Hypothetical protein Cj1162c	P	-27.41
Cj1163c		Cobalt/zinc/cadmium resistance protein CzcD	P	-24.40
Cj1164c		hypothetical protein	S	-0.15
Cj1166c		Membrane protein	S	-25.93
Cj1167	ldh	L-lactate dehydrogenase (EC 1.1.1.27)	C	-1.56
Cj1168c		putative integral membrane protein (dedA homolog)	S	-25.60
Cj1170c	omp50	Membrane protein	S	-30.56
Cj1171c	ppi	Peptidyl-prolyl cis-trans isomerase (EC 5.2.1.8)	O	-0.69
Cj1172c		Probable transcriptional regulatory protein YebC	K	-2.45
Cj1175c	argS	Arginyl-tRNA synthetase (EC 6.1.1.19)	J	0.45
Cj1176c	tatA	Twin-arginine translocation protein TatA	U	-0.33
Cj1178c		Highly acidic protein	A	-0.88
Cj1180c		ABC transporter, ATP-binding protein	V	-24.26
Cj1181c	tsf	Translation elongation factor Ts	J	1.12
Cj1182c	rpsB	SSU ribosomal protein S2p (SAe)	J	-0.85
Cj1183c	cfa	Putative cyclopropane-fatty-acyl-phospholipid synthase (EC 2.1.1.79)	M	-3.75
Cj1184c	petC	Ubiquinol-cytochrome C reductase, cytochrome C1 subunit	C	-2.11
Cj1185c	petB	Ubiquinol-cytochrome C reductase, cytochrome B subunit (EC 1.10.2.2)	C	-2.45
Cj1186c	petA	Ubiquinol-cytochrome C reductase iron-sulfur subunit (EC 1.10.2.2)	C	-4.36
Cj1187c	arsB	putative anion permease	P	-24.65
Cj1188c	gidA	tRNA-5-carboxymethylaminomethyl-2-thiouridine(34) synthesis protein MnmG	D	1.79
Cj1189c	cetB	Signal transduction protein CetB, mediates an energy taxis response	KT	-1.31
Cj1190c	cetA	Signal transduction protein CetA, mediates an energy taxis response	NT	-1.32
Cj1191c		Signal transduction protein CetB, mediates an energy taxis response	KT	-4.20
Cj1192	dctA	Putative C4-dicarboxylate transport protein	U	-1.86
Cj1193c		Putative periplasmic protein	-	30.05
Cj1194		Probable low-affinity inorganic phosphate transporter	U	-1.55
Cj1195c	pyrC2	Amidohydrolase	F	0.52
Cj1196c	gpsA	Glycerol-3-phosphate dehydrogenase [NAD(P)+] (EC 1.1.1.94)	I	-1.21

Cj1197c	gatB	Aspartyl-tRNA(Asn) amidotransferase subunit B (EC 6.3.5.6) @ Glutamyl-tRNA(Gln) amidotransferase subunit B (EC 6.3.5.7)	J	-1.66
Cj1198	luxS	S-ribosylhomocysteine lyase (EC 4.4.1.21) @ Autoinducer-2 production protein LuxS	H	-0.31
Cj1199		2-Oxobutyrate oxidase, putative	C	4.82
Cj1200		Methionine ABC transporter substrate-binding protein	P	-1.00
Cj1201	metE	5-methyltetrahydropteroyltriglutamate--homocysteine methyltransferase (EC 2.1.1.14)	E	0.98
Cj1204c	atpB	ATP synthase F0 sector subunit a (EC 3.6.3.14)	C	-2.65
Cj1205c	radA	DNA repair protein RadA	O	-1.79
Cj1206c	ftsY	Signal recognition particle receptor FtsY	U	-28.20
Cj1207c		Putative lipoprotein thiredoxin	CO	-0.14
Cj1209		Ribonuclease Y	S	-1.23
Cj1210		Membrane protein	S	-0.92
Cj1211		DNA transfer protein	S	26.67
Cj1213c	glcD	(S)-2-hydroxy-acid oxidase (EC 1.1.3.15)	C	-0.22
Cj1214c		hypothetical protein	C	-29.62
Cj1215		Putative membrane protein related to metalloendopeptidases	M	0.48
Cj1217c		FIG00003370: Multicopper polyphenol oxidase	S	-0.96
Cj1218c	ribA	Riboflavin synthase eubacterial/eukaryotic (EC 2.5.1.9)	H	26.44
Cj1219c		Putative periplasmic protein	S	0.86
Cj1220	groES	Heat shock protein 10 kDa family chaperone GroES	O	-1.31
Cj1221	groEL	Heat shock protein 60 kDa family chaperone GroEL	O	-1.32
Cj1222c	dccS	Two-component system histidine kinase DccS	T	-0.26
Cj1223c	dccR	Two-component system response regulator DccR	KT	-29.92
Cj1224		Hemerythrin domain protein	P	-2.66
Cj1225		Ribbon-helix-helix protein, copG family domain protein	S	-0.66
Cj1226c		Putative two-component sensor	T	-3.30
Cj1227c		Putative two-component regulator	K	-1.19
Cj1228c	htrA	HtrA protease/chaperone protein / Serine protease (Protease DO) (EC 3.4.21.-)	M	-1.12
Cj1229	cbpA	DnaJ-class molecular chaperone CbpA	O	-29.86
Cj1230	hspR	HspR, transcriptional repressor of DnaK operon	K	2.02
Cj1233		Predicted phosphatase	S	0.46
Cj1234	glyS	Glycyl-tRNA synthetase beta chain (EC 6.1.1.14)	J	-0.71
Cj1235		Membrane protein related to metalloendopeptidases	M	-3.27
Cj1236		SAM-dependent methyltransferase, MidA	S	1.74
Cj1237c		Putative phosphatase	FP	-2.07
Cj1238	pdxJ	Pyridoxine 5'-phosphate synthase (EC 2.6.99.2)	H	-28.68
Cj1239	pdxA	4-hydroxythreonine-4-phosphate dehydrogenase (EC 1.1.1.262)	H	-3.27
Cj1240c		hypothetical protein	-	-29.49
Cj1241		Putative transmembrane transport protein	EGP	-28.14
Cj1242		hypothetical protein	-	-23.59
Cj1243	hemE	Uroporphyrinogen III decarboxylase (EC 4.1.1.37)	H	-0.75
Cj1244		Putative Fe-S oxidoreductase	C	-2.17

Cj1245c		Membrane protein	-	-29.13
Cj1246c	uvrC	Excinuclease ABC subunit C	L	-0.41
Cj1247c		hypothetical protein	-	-25.64
Cj1248	guaA	GMP synthase [glutamine-hydrolyzing], amidotransferase subunit (EC 6.3.5.2) / GMP synthase [glutamine-hydrolyzing], ATP pyrophosphatase subunit (EC 6.3.5.2)	F	-2.94
Cj1249		hypothetical protein	-	2.68
Cj1250	purD	Phosphoribosylamine--glycine ligase (EC 6.3.4.13)	F	0.32
Cj1251		hypothetical protein	S	0.00
Cj1252		LPS-assembly protein LptD @ Organic solvent tolerance protein precursor	M	2.85
Cj1253	pnp	Polyribonucleotide nucleotidyltransferase (EC 2.7.7.8)	J	0.59
Cj1256c		Membrane protein	S	-1.47
Cj1258		Low molecular weight protein tyrosine phosphatase (EC 3.1.3.48)	T	-1.54
Cj1259	porA	Major outer membrane protein	P	0.56
Cj1260c	dnaJ	Chaperone protein DnaJ	O	-2.15
Cj1261	racR	Two-component system response regulator RacR	K	3.63
Cj1262	racS	Two-component system histidine kinase RacS	T	-2.17
Cj1263	recR	Recombination protein RecR	L	-1.65
Cj1264c	hydD	Hydrogenase maturation protease	C	-28.06
Cj1265c	hydC	Quinone-reactive Ni/Fe hydrogenase, cytochrome b subunit	C	-3.71
Cj1266c	hydB	Quinone-reactive Ni/Fe-hydrogenase large chain (EC 1.12.5.1)	C	-0.53
Cj1267c	hydA	Quinone-reactive Ni/Fe-hydrogenase small chain (EC 1.12.5.1)	C	-1.39
Cj1268c	mnmC	tRNA (5-methylaminomethyl-2-thiouridylate)-methyltransferase (EC 2.1.1.61) / FAD-dependent cmnm(5)s(2)U34 oxidoreductase	J	-3.57
Cj1269c	amiA	N-acetylmuramoyl-L-alanine amidase (EC 3.5.1.28)	M	-0.74
Cj1270c		UFA synthesis enzyme FabX, enoyl-[ACP] 2,3-dehydrogenase/trans-2-decenoyl-[ACP] isomerase	S	-1.34
Cj1271c	tyrS	Tyrosyl-tRNA synthetase (EC 6.1.1.1)	J	-3.42
Cj1272c	spoT	Guanosine-3',5'-bis(diphosphate) 3'-pyrophosphohydrolase (EC 3.1.7.2) / GTP pyrophosphokinase (EC 2.7.6.5), (p)ppGpp synthetase II	KT	-2.29
Cj1274c	pyrH	Uridylate kinase (EC 2.7.4.22)	F	-2.76
Cj1275c		Murein hydrolase activator EnvC	M	4.54
Cj1277c		Cell-division-associated, ABC-transporter-like signaling protein FtsE	D	-29.79
Cj1278c	trmB	tRNA (guanine(46)-N(7))-methyltransferase (EC 2.1.1.33)	J	0.34
Cj1279c		Putative fibronectin domain-containing lipoprotein	S	1.27
Cj1280c		LSU rRNA pseudouridine(1911/1915/1917) synthase (EC 5.4.99.23)	J	3.57
Cj1282	mrdB	Rod shape-determining protein RodA	D	0.00
Cj1284	ktrA	KtrAB potassium uptake system, peripheral membrane component KtrA	P	-1.55
Cj1285c		Chorismate dehydratase (EC 4.2.1.151)	H	2.29

Cj1286c	upp	Uracil phosphoribosyltransferase (EC 2.4.2.9)	F	1.40
Cj1287c		NADP-dependent malic enzyme (EC 1.1.1.40)	C	-3.53
Cj1288c	gltX	Glutamyl-tRNA synthetase (EC 6.1.1.17)	J	-1.71
Cj1289		Putative periplasmic protein	O	0.26
Cj1290c	accC	Biotin carboxylase of acetyl-CoA carboxylase (EC 6.3.4.14)	I	-0.48
Cj1291c	accB	Biotin carboxyl carrier protein of acetyl-CoA carboxylase	I	-3.94
Cj1292	dcd	Deoxycytidine triphosphate deaminase (EC 3.5.4.13)	F	-1.12
Cj1293	pseB	UDP-N-acetylglucosamine 4,6-dehydratase (inverting) (EC 4.2.1.115)	M	-2.76
Cj1294	pseC	UDP-4-amino-4,6-dideoxy-N-acetyl-beta-L-altrosamine transaminase (EC 2.6.1.92)	E	-2.85
Cj1295		Protein containing aminopeptidase domain	Q	27.41
Cj1297		hypothetical protein	V	-25.90
Cj1298		GDP-4-amino-4,6-dideoxy-alpha-D-acetylglucosamine N-acetyltransferase	V	0.00
Cj1299	acpP2	hypothetical protein	IQ	-1.57
Cj1300		hypothetical protein	H	-1.45
Cj1301		hypothetical protein	E	-31.73
Cj1302		HAD-superfamily phosphatase, subfamily IIIC	Q	-1.28
Cj1303	fabH2	3-oxoacyl-[acyl-carrier-protein] synthase, KASIII (EC 2.3.1.180)	I	-6.37
Cj1304	acpP3	hypothetical protein	IQ	-25.96
Cj1305c		hypothetical protein	E	-2.22
Cj1306c		hypothetical protein	E	5.23
Cj1307		Putative amino acid activating enzyme (EC 6.3.2.-)	Q	-1.10
Cj1308		hypothetical protein	IQ	-0.60
Cj1309c		hypothetical protein	-	-2.10
Cj1310c		hypothetical protein	E	-0.58
Cj1311	pseF	Pseudaminic acid cytidylyltransferase (EC 2.7.7.81)	M	2.42
Cj1312	pseG	UDP-6-deoxy-AltdiNAc hydrolase (PseG, third step of pseudaminic acid biosynthesis)	M	-27.16
Cj1313	pseH	UDP-4-amino-4,6-dideoxy-N-acetyl-beta-L-altrosamine N-acetyltransferase (EC 2.3.1.202)	J	35.25
Cj1314c	hisF	Similar to imidazole glycerol phosphate synthase cyclase subunit (LPS cluster)	E	11.51
Cj1315c	hisH	Similar to imidazole glycerol phosphate synthase amidotransferase subunit (LPS cluster)	E	-1.85
Cj1316c	pseA	Pseudaminic acid biosynthesis protein PseA, possible Pse5Ac7Ac acetamidino synthase	D	-2.06
Cj1317	pseI	Pseudaminic acid synthase (EC 2.5.1.97)	M	0.24
Cj1319		GDP-N-acetylglucosamine 4,6-dehydratase [NAD+]	M	2.58
Cj1320		GDP-2-acetamido-2,6-dideoxy-alpha-D-xylo-hexos-4-ulose aminotransferase [PLP]	E	-26.49
Cj1322		hypothetical protein	I	-0.64
Cj1323		hypothetical protein	I	0.00
Cj1324		Legionaminic acid biosynthesis protein PtmG	-	0.33
Cj1325		hypothetical protein	Q	-0.07

Cj1327	neuB2	N,N'-diacetyllegionaminic acid synthase (EC 2.5.1.101)	M	-0.07
Cj1328	neuC2	UDP-N,N'-diacetylbacillosamine 2-epimerase (hydrolyzing) (EC 3.2.1.184)	M	1.10
Cj1329		Glucosamine-1-phosphate guanylyltransferase	M	-29.20
Cj1330		Glutamine--fructose-6-phosphate transaminase (isomerizing), isomerase subunit (EC 2.6.1.16)	S	-2.26
Cj1331	ptmB	CMP-N,N'-diacetyllegionaminic acid synthase (EC 2.7.7.82)	M	-0.63
Cj1332	ptmA	Glutamine--fructose-6-phosphate transaminase (isomerizing), glutaminase subunit (EC 2.6.1.16)	N	-2.35
Cj1333	pseD	Motility accessory factor	S	-0.27
Cj1334	maf3	Motility accessory factor	S	-2.52
Cj1337	pseE	Motility accessory factor	S	-1.14
Cj1338c	flaB	Flagellin	N	0.69
Cj1339c	flaA	Flagellin	N	-0.57
Cj1340c		Motility accessory factor	S	-0.70
Cj1341c	maf6	Motility accessory factor	S	-0.03
Cj1342c	maf7	hypothetical protein	E	-2.65
Cj1343c		Type II secretion envelope pseudopilin protein (PulG,guides folded protein to PulD in outer membrane)	NU	0.00
Cj1344c		N(6)-L-threonylcarbamoyladenine synthase (EC 2.3.1.234)	J	-30.00
Cj1345c		Putative periplasmic protein	S	0.22
Cj1346c	dxr	1-deoxy-D-xylulose 5-phosphate reductoisomerase (EC 1.1.1.267)	I	4.61
Cj1347c	cdsA	Phosphatidate cytidylyltransferase (EC 2.7.7.41)	S	-1.60
Cj1348c		Putative coiled-coil protein	-	25.30
Cj1349c		Fibronectin/fibrinogen-binding protein	K	1.05
Cj1350	mobA	Molybdenum cofactor guanylyltransferase (EC 2.7.7.77)	H	-25.21
Cj1351	pldA	Phospholipase A1 (EC 3.1.1.32) (EC 3.1.1.4) @ Outer membrane phospholipase A	M	23.06
Cj1353	ceuC	hypothetical protein	P	2.58
Cj1354	ceuD	hypothetical protein	P	-26.47
Cj1355	ceuE	Enterochelin uptake periplasmic binding protein	P	-1.17
Cj1356c		Integral membrane protein	S	0.96
Cj1357c	nrfA	Cytochrome c552 precursor (EC 1.7.2.2)	C	-1.76
Cj1358c	nrfH	Cytochrome c nitrite reductase, small subunit NrfH	C	28.91
Cj1359	ppk	Polyphosphate kinase (EC 2.7.4.1)	F	-0.63
Cj1361c		hypothetical protein	T	3.87
Cj1362	ruvB	Holliday junction ATP-dependent DNA helicase RuvB (EC 3.6.4.12)	L	-4.08
Cj1363	amaA	Acid membrane antigen A	S	0.00
Cj1364c	fumC	Fumarate hydratase class II (EC 4.2.1.2)	C	-0.67
Cj1365c		Putative secreted serine protease (EC 3.4.21.-)	O	-0.13
Cj1366c	glmS	Glutamine--fructose-6-phosphate aminotransferase [isomerizing] (EC 2.6.1.16)	M	-0.96
Cj1367c		[Protein-PII] uridylyltransferase (EC 2.7.7.59) / [Protein-PII]-UMP uridylyl-removing enzyme	O	-1.06
Cj1368		Aminodeoxyfutalosine synthase (EC 2.5.1.120)	H	-2.13

Cj1370		Xanthine-guanine phosphoribosyltransferase (EC 2.4.2.22)	F	-2.57
Cj1371		Outer-membrane-phospholipid-binding lipoprotein MlaA	M	0.25
Cj1372		Phospholipid ABC transporter shuttle protein MlaC	Q	-0.57
Cj1373		Exporter protein, RND family	S	0.00
Cj1374c		Nucleoside 5-triphosphatase RdgB (dHAPTP, dITP, XTP-specific) (EC 3.6.1.66)	F	3.84
Cj1375		Uncharacterized MFS-type transporter	EGP	1.12
Cj1376		hypothetical protein	S	-31.76
Cj1377c		Iron-sulfur cluster-binding protein	C	-3.23
Cj1378	selA	L-seryl-tRNA(Sec) selenium transferase (EC 2.9.1.1)	J	-0.65
Cj1379	selB	Selenocysteine-specific translation elongation factor	J	4.75
Cj1380		Putative periplasmic protein	O	-0.39
Cj1381		putative lipoprotein	-	24.58
Cj1382c	fldA	Flavodoxin 1	C	-0.23
Cj1383c		hypothetical protein	-	-26.14
Cj1384c		hypothetical protein	S	2.76
Cj1385	katA	Catalase KatE (EC 1.11.1.6)	C	0.51
Cj1386		Ankyrin-repeat containing protein	S	0.01
Cj1388		RidA/YER057c/UK114 superfamily protein	J	0.00
Cj1393	metC'	Cystathionine beta-lyase (EC 4.4.1.8)	E	-8.23
Cj1394		Formerly called adenylosuccinate lyase (similar to archaeal version), but most likely something else	F	-3.36
Cj1397		Ferrous iron transporter-associated protein FeoA	P	0.00
Cj1398	feoB	Ferrous iron transporter FeoB	P	-2.84
Cj1399c	hydA2	hydrogenase, (NiFe)/(NiFeSe) small subunit family	C	-2.06
Cj1400c	fabI	Enoyl-[acyl-carrier-protein] reductase [NADH] (EC 1.3.1.9)	I	-1.11
Cj1401c	tpiA	Triosephosphate isomerase (EC 5.3.1.1)	G	-3.03
Cj1402c	pgk	Phosphoglycerate kinase (EC 2.7.2.3)	F	0.04
Cj1403c	gapA	NAD-dependent glyceraldehyde-3-phosphate dehydrogenase (EC 1.2.1.12)	G	-1.60
Cj1404	nadD	Nicotinate-nucleotide adenylyltransferase (EC 2.7.7.18)	H	0.00
Cj1405		Ribosomal silencing factor RsfA	J	-0.35
Cj1406c		Putative periplasmic protein	S	-1.71
Cj1407c		Phosphoglucomutase (EC 5.4.2.2) @ Phosphomannomutase (EC 5.4.2.8)	G	-0.25
Cj1408	fliL	Flagellar basal body-associated protein FliL	N	-3.32
Cj1409	acpS	Holo-[acyl-carrier-protein] synthase (EC 2.7.8.7)	I	-0.48
Cj1410c		Membrane protein	-	-26.13
Cj1411c		Cytochrome P450 family protein	C	-2.70
Cj1412c		Probable integral membrane protein Cj1412c	S	0.55
Cj1413c	kpsS	Capsular polysaccharide export system protein KpsS	M	-26.38
Cj1414c	kpsC	Capsular polysaccharide export system protein KpsC	M	2.53
Cj1415c	cysC	Cytidine diphosphoramidate kinase Cj1415	P	-29.06
Cj1416c		Phosphoglutamine cytidylyltransferase (EC 2.7.7.-) in O-methyl phosphoramidate capsule modification	M	-1.20
Cj1417c		gamma-Glutamyl-CDP-amidate hydrolase involved in O-methyl phosphoramidate capsule modification	S	-23.42

Cj1418c		L-glutamine kinase involved in O-methyl phosphoramidate capsule modification	G	-0.05
Cj1419c		Methyltransferase (EC 2.1.1.-), possibly involved in O-methyl phosphoramidate capsule modification	Q	4.62
Cj1420c		Methyltransferase (EC 2.1.1.-), possibly involved in O-methyl phosphoramidate capsule modification	Q	-1.63
Cj1421c		Possible sugar transferase	S	-2.48
Cj1422c		hypothetical protein	S	-5.72
Cj1423c	hddC	D-glycero-alpha-D-manno-heptose 1-phosphate guanylyltransferase (EC 2.7.7.71)	JM	25.59
Cj1424c	gmhA2	D-sedoheptulose 7-phosphate isomerase (EC 5.3.1.28)	G	-0.01
Cj1425c	hddA	D-glycero-alpha-D-manno-heptose 7-phosphate kinase (EC 2.7.1.168)	S	1.31
Cj1426c		hypothetical protein	J	-2.00
Cj1427c		UDP-glucose 4-epimerase (EC 5.1.3.2)	M	-0.98
Cj1428c	fcl	GDP-L-fucose synthetase (EC 1.1.1.271)	GM	-0.92
Cj1429c		hypothetical protein	S	23.07
Cj1430c	rfbC	dTDP-4-dehydrorhamnose 3,5-epimerase (EC 5.1.3.13)	M	-2.74
Cj1431c	hddC	hypothetical protein	S	-1.81
Cj1432c		hypothetical protein	M	-0.34
Cj1433c		hypothetical protein	H	-3.57
Cj1434c		Putative sugar transferase	S	0.09
Cj1435c		hypothetical protein	E	-1.77
Cj1436c		hypothetical protein	E	-1.14
Cj1437c		hypothetical protein	E	-3.58
Cj1438c		Putative sugar transferase	S	-1.39
Cj1439c	glf	UDP-galactopyranose mutase (EC 5.4.99.9)	M	1.87
Cj1440c		Beta-1,3-galactosyltransferase / Beta-1,4-galactosyltransferase	M	-0.71
Cj1441c	kfiD	UDP-glucose 6-dehydrogenase (EC 1.1.1.22)	C	-2.80
Cj1442c		Predicted glycosyltransferase involved in capsule biosynthesis	M	-2.63
Cj1443c	kpsF	D-arabinose-5-phosphate isomerase (EC 5.3.1.13)	M	-2.90
Cj1444c	kpsD	Capsular polysaccharide export system periplasmic protein KpsD	M	-0.65
Cj1445c	kpsE	Capsular polysaccharide export system inner membrane protein KpsE	M	0.53
Cj1447c	kpsT	Capsular polysaccharide ABC transporter, ATP-binding protein KpsT	GM	3.58
Cj1449c		Hypothetical UPF0306 protein Cj1449c	S	-25.78
Cj1450		ATP/GTP-binding protein	-	0.90
Cj1451	dut	Dimeric dUTPase (EC 3.6.1.23)	S	-0.26
Cj1452		Probable integral membrane protein Cj1452	S	-27.67
Cj1453c	tilS	tRNA(Ile)-lysidine synthetase (EC 6.3.4.19)	J	0.45
Cj1454c		Ribosomal protein S12p Asp88 (E. coli) methylthiotransferase (EC 2.8.4.4)	J	1.25
Cj1455	prfB	Peptide chain release factor 2	J	-0.30

Cj1456c		Putative periplasmic protein	-	0.00
Cj1457c	truD	tRNA pseudouridine(13) synthase (EC 5.4.99.27)	J	-2.02
Cj1458c	thiL	Thiamine-monophosphate kinase (EC 2.7.4.16)	H	27.86
Cj1459		hypothetical protein	C	-1.85
Cj1460		JHP0747 family	-	-31.27
Cj1461		16S rRNA (guanine(966)-N(2))-methyltransferase (EC 2.1.1.171)	L	0.00
Cj1462	flgI	Flagellar P-ring protein FlgI	N	2.20
Cj1463	flgJ	hypothetical protein	MNO	1.80
Cj1464	flgM	hypothetical protein	S	0.00
Cj1465		hypothetical protein	-	0.10
Cj1466	flgK	Flagellar hook-associated protein FlgK	N	0.61
Cj1467		hypothetical protein	L	0.00
Cj1471c	ctsE	Type II secretion cytoplasmic ATP binding protein (PulE, ATPase)	NU	1.55
Cj1472c		Transformation system protein	-	1.76
Cj1473c	ctsP	Transformation system protein	S	-23.14
Cj1474c	ctsD	Type II secretion outermembrane pore forming protein (PulD)	NU	-25.58
Cj1476c		Pyruvate-flavodoxin oxidoreductase	C	-1.53
Cj1477c		Haloacid dehalogenase-like hydrolase	S	0.38
Cj1478c	cadF	Outer membrane fibronectin-binding protein	M	-1.62
Cj1479c	rpsI	SSU ribosomal protein S9p (S16e)	J	-1.19
Cj1480c	rplM	LSU ribosomal protein L13p (L13Ae)	J	-0.62
Cj1481c		Helicase	L	0.39
Cj1482c		Helicase / RecB family exonuclease	L	-0.40
Cj1484c		Membrane protein	-	-24.13
Cj1486c		Putative periplasmic protein	S	0.00
Cj1487c	ccoP	Cytochrome c oxidase (cbb3-type) subunit CcoP (EC 1.9.3.1)	C	-0.80
Cj1489c	ccoO	Cytochrome c oxidase (cbb3-type) subunit CcoO (EC 1.9.3.1)	C	-0.68
Cj1490c	ccoN	Cytochrome c oxidase (cbb3-type) subunit CcoN (EC 1.9.3.1)	C	-1.91
Cj1491c		Two-component system response regulator protein	T	-1.35
Cj1492c		Two-component system sensor histidine kinase	T	-2.87
Cj1494c	carA	Carbamoyl-phosphate synthase small chain (EC 6.3.5.5)	F	-3.31
Cj1495c		Hypothetical protein probably associated with Carbamoyl-phosphate synthase	S	-0.91
Cj1496c		Putative periplasmic protein	S	-0.18
Cj1498c	purA	Adenylosuccinate synthetase (EC 6.3.4.4)	F	-1.82
Cj1500		UPF0394 inner membrane protein YedE	S	20.08
Cj1503c	putA	Proline dehydrogenase (EC 1.5.5.2) / Delta-1-pyrroline-5-carboxylate dehydrogenase (EC 1.2.1.88)	C	-0.85
Cj1504c	selD	Selenide,water dikinase (EC 2.7.9.3) @ selenocysteine-containing	E	-27.46
Cj1505c		Hypothetical protein Cj1505c	O	0.01

Cj1506c		Methyl-accepting chemotaxis protein	NT	-0.58
Cj1507c		N-terminal HTH domain of molybdenum-binding protein family	S	1.38
Cj1508c	fdhD	Sulfur carrier protein FdhD	C	-0.64
Cj1509c	fdhC	Putative formate dehydrogenase, cytochrome B subunit (EC 1.2.1.2)	C	0.00
Cj1510c	fdhB	Formate dehydrogenase-O, iron-sulfur subunit (EC 1.2.1.2); Putative formate dehydrogenase iron-sulfur subunit (EC 1.2.1.2)	C	-2.05
Cj1511c	fdhA	Formate dehydrogenase-O, major subunit (EC 1.2.1.2) @ selenocysteine-containing	C	-2.22
Cj1514c		Putative formate dehydrogenase-specific chaperone	S	-2.62
Cj1515c		Carboxynorspermidine decarboxylase (EC 4.1.1.96)	H	-1.40
Cj1516		Laccase (EC 1.10.3.2)	Q	-0.41
Cj1518	moaE	Molybdopterin synthase catalytic subunit MoaE (EC 2.8.1.12)	H	-29.28
Cj1519	moeA2	Molybdopterin molybdenumtransferase (EC 2.10.1.1)	H	0.48
Cj1521c		CRISPR-associated protein Cas2	L	-28.44
Cj1522c		CRISPR-associated protein Cas1	L	-0.93
Cj1523c		CRISPR-associated endonuclease Cas9	L	-0.95
Cj1529c	purM	Phosphoribosylformylglycinamidine cyclo-ligase (EC 6.3.3.1)	F	-2.16
Cj1530	coaE	Dephospho-CoA kinase (EC 2.7.1.24)	F	-5.49
Cj1531	dapF	Diaminopimelate epimerase (EC 5.1.1.7)	E	-27.64
Cj1532		Putative periplasmic protein	S	1.16
Cj1533c		Putative helix-turn-helix containsing protein	S	1.93
Cj1534c		DNA protection during starvation protein	P	-1.97
Cj1535c	pgi	Glucose-6-phosphate isomerase (EC 5.3.1.9)	G	0.38
Cj1536c	galU	UTP--glucose-1-phosphate uridylyltransferase (EC 2.7.7.9)	M	-0.60
Cj1537c	acs	Acetyl-CoA synthetase (EC 6.2.1.1)	F	-1.05
Cj1540		Tungstate ABC transporter, substrate-binding protein	H	-0.39
Cj1541		Lactam utilization protein LamB	S	0.14
Cj1542		Allophanate hydrolase 2 subunit 1 (EC 3.5.1.54)	E	5.49
Cj1543		Allophanate hydrolase 2 subunit 2 (EC 3.5.1.54)	E	-2.91
Cj1544c		Membrane protein	EG	-26.21
Cj1545c		MdaB protein homolog	S	-1.06
Cj1546		Transcriptional regulator, HxlR family	K	27.18
Cj1547		hypothetical protein	M	1.63
Cj1548c		Cinnamyl alcohol dehydrogenase/reductase (EC 1.1.1.195) @ Alcohol dehydrogenase (EC 1.1.1.1)	C	-1.68
Cj1549c	hsdR	Type I restriction-modification system, restriction subunit R (EC 3.1.21.3)	V	-1.99
Cj1550c	rloH	ATP/GTP-binding protein	L	-2.21
Cj1551c	hsdS	Type I restriction-modification system, specificity subunit S	V	1.46
Cj1552c	mloB	Predicted transcriptional regulator containing an HTH domain and an uncharacterized domain shared with the mammalian protein Schlafen	K	-27.26

Cj1553c	hsdM	Type I restriction-modification system, DNA-methyltransferase subunit M (EC 2.1.1.72)	V	3.67
Cj1555c		Rrf2-linked NADH-flavin reductase	S	-7.11
Cj1563c		Transcriptional regulator	K	-23.43
Cj1564		Methyl-accepting chemotaxis signal transduction protein	NT	-1.38
Cj1565c	pflA	Paralysed flagella protein PflA	N	-2.01
Cj1567c	nuoM	NADH-ubiquinone oxidoreductase chain M (EC 1.6.5.3)	C	-3.59
Cj1568c	nuoL	NADH-ubiquinone oxidoreductase chain L (EC 1.6.5.3)	CP	-29.46
Cj1569c	nuoK	NADH-ubiquinone oxidoreductase chain K (EC 1.6.5.3)	C	0.00
Cj1571c	nuoI	NADH-ubiquinone oxidoreductase chain I (EC 1.6.5.3)	C	-2.89
Cj1572c	nuoH	NADH-ubiquinone oxidoreductase chain H (EC 1.6.5.3)	C	-24.09
Cj1573c	nuoG	NADH-ubiquinone oxidoreductase chain G (EC 1.6.5.3)	C	0.12
Cj1574c		FIG00010922: Possible NADH-ubiquinone oxidoreductase subunit	C	0.49
Cj1575c		NADH-ubiquinone oxidoreductase chain E (EC 1.6.5.3)	S	-29.42
Cj1576c	nuoD	NADH-ubiquinone oxidoreductase chain D (EC 1.6.5.3)	C	-1.63
Cj1577c	nuoC	NADH-ubiquinone oxidoreductase chain C (EC 1.6.5.3)	C	-0.29
Cj1578c	nuoB	NADH-ubiquinone oxidoreductase chain B (EC 1.6.5.3)	C	-3.07
Cj1579c	nuoA	NADH ubiquinone oxidoreductase chain A (EC 1.6.5.3)	C	-26.56
Cj1580c		ABC transporter, ATP-binding protein (cluster 5, nickel/peptides/opines)	E	0.00
Cj1583c		Putative peptide ABC-transport system permease protein	P	0.00
Cj1584c		ABC transporter, substrate-binding protein (cluster 5, nickel/peptides/opines)	E	-1.46
Cj1585c		Predicted D-lactate dehydrogenase, Fe-S protein, FAD/FMN-containing	C	-3.72
Cj1586	cgb	Putative bacterial haemoglobin	C	-2.51
Cj1587c		ABC-type siderophore export system, fused ATPase and permease components	V	-3.23
Cj1589		MBL-fold metallo-hydrolase superfamily	S	0.00
Cj1590	infA	Translation initiation factor 1	J	-0.65
Cj1592	rpsM	SSU ribosomal protein S13p (S18e)	J	2.12
Cj1593	rpsK	SSU ribosomal protein S11p (S14e)	J	-2.56
Cj1594	rpsD	SSU ribosomal protein S4p (S9e) @ SSU ribosomal protein S4p (S9e), zinc-independent	J	-0.61
Cj1595	rpoA	DNA-directed RNA polymerase alpha subunit (EC 2.7.7.6)	K	-0.20
Cj1596	rplQ	LSU ribosomal protein L17p	J	-4.18
Cj1597	hisG	ATP phosphoribosyltransferase (EC 2.4.2.17) => HisGl	F	-0.45
Cj1598	hisD	Histidinol dehydrogenase (EC 1.1.1.23)	E	-0.52
Cj1599	hisB	Histidinol-phosphatase (EC 3.1.3.15) / Imidazoleglycerol-phosphate dehydratase (EC 4.2.1.19)	E	-1.52
Cj1600	hisH	Imidazole glycerol phosphate synthase amidotransferase subunit HisH	E	-28.89
Cj1601	hisA	Phosphoribosylformimino-5-aminoimidazole carboxamide ribotide isomerase (EC 5.3.1.16)	E	-2.00
Cj1602		hypothetical protein	S	0.63
Cj1603	hisF	Imidazole glycerol phosphate synthase cyclase subunit	E	-2.13

Cj1604	hisI	Phosphoribosyl-AMP cyclohydrolase (EC 3.5.4.19) / Phosphoribosyl-ATP pyrophosphatase (EC 3.6.1.31)	E	-1.97
Cj1605c	dapD	2,3,4,5-tetrahydropyridine-2,6-dicarboxylate N-succinyltransferase (EC 2.3.1.117)	E	-0.94
Cj1606c	mrp	putative ATP/GTP-binding protein (mrp protein homolog)	D	-1.80
Cj1607	ispDF	2-C-methyl-D-erythritol 4-phosphate cytidylyltransferase (EC 2.7.7.60) / 2-C-methyl-D-erythritol 2,4-cyclodiphosphate synthase (EC 4.6.1.12)	I	-3.01
Cj1608		Possible two-component regulator	T	-0.63
Cj1609		Sulfate adenylyltransferase (EC 2.7.7.4)	P	-1.20
Cj1611	rpsT	SSU ribosomal protein S20p	J	-21.99
Cj1612	prfA	Peptide chain release factor 1	J	2.79
Cj1613c		Putative heme oxygenase	P	-0.81
Cj1614	chuA	Haemin uptake system outer membrane receptor	P	-26.07
Cj1615	chuB	Haemin uptake system permease protein	P	-27.44
Cj1616	chuC	Haemin uptake system ATP-binding protein	HP	-9.31
Cj1617	chuD	Haemin uptake system periplasmic haemin-binding protein	P	0.00
Cj1618c		hypothetical protein	S	26.86
Cj1619	kgtP	Alpha-ketoglutarate permease	EGP	-4.44
Cj1620c	mutY	A/G-specific adenine glycosylase (EC 3.2.2.-)	L	1.20
Cj1621		Putative periplasmic protein	S	0.66
Cj1622	ribD	Diaminohydroxyphosphoribosylaminopyrimidine deaminase (EC 3.5.4.26) / 5-amino-6-(5-phosphoribosylamino)uracil reductase (EC 1.1.1.193)	H	-3.22
Cj1623		Membrane protein	-	23.97
Cj1624c	sdaA	L-serine dehydratase, beta subunit (EC 4.3.1.17) / L-serine dehydratase, alpha subunit (EC 4.3.1.17)	E	-0.59
Cj1625c	sdaC	Serine transporter	E	-0.86
Cj1626c		Putative periplasmic protein	S	2.19
Cj1627c		hypothetical protein	S	-30.43
Cj1630	tonB2	putative TonB-dependent receptor	U	-1.39
Cj1631c		hypothetical protein	-	3.36
Cj1632c		hypothetical protein	-	0.00
Cj1633		Possible RNA methyltransferase aq_898	D	-1.03
Cj1634c	aroC	Chorismate synthase (EC 4.2.3.5)	E	-0.85
Cj1635c	rnc	Ribonuclease III (EC 3.1.26.3)	J	-28.82
Cj1636c	rnhA	Ribonuclease HI (EC 3.1.26.4)	L	-24.43
Cj1637c		Putative periplasmic protein	G	-27.11
Cj1638	dnaG	DNA primase DnaG	L	2.14
Cj1639		NifU protein homolog	O	-6.06
Cj1641	murE	UDP-N-acetylmuramoyl-dipeptide--2,6-diaminopimelate ligase (EC 6.3.2.13)	M	-3.11
Cj1642		Nucleoid-associated protein YaaK	S	-25.97
Cj1643		Putative periplasmic protein	M	0.48
Cj1644	ispA	(2E,6E)-farnesyl diphosphate synthase (EC 2.5.1.10)	H	0.19
Cj1645	tkt	Transketolase (EC 2.2.1.1)	G	-0.23
Cj1646	iamB	ABC transporter, permease protein (cluster 9, phospholipid)	Q	0.00

Cj1647	iamA	ABC transporter, ATP-binding protein (cluster 9, phospholipid)	Q	-0.52
Cj1648		ABC transporter, substrate-binding protein (cluster 9, phospholipid)	Q	-0.40
Cj1649		putative lipoprotein	S	1.40
Cj1651c	map	Methionine aminopeptidase (EC 3.4.11.18)	E	-1.57
Cj1652c	murI	Glutamate racemase (EC 5.1.1.3)	M	-30.10
Cj1653c		Probable lipoprotein nlpC precursor	M	-3.36
Cj1658		High-affinity Fe2+/Pb2+ permease precursor	P	-1.05
Cj1659	p19	Periplasmic protein p19 involved in high-affinity Fe2+ transport	P	0.55
Cj1660		Fe2+ ABC transporter, substrate binding protein	S	0.00
Cj1661		Fe2+ ABC transporter, permease protein 1	V	-23.75
Cj1662		Fe2+ ABC transporter, permease protein 2	V	-0.37
Cj1663		Fe2+ ABC transporter, ATP-binding subunit	V	-1.24
Cj1664		Possible periplasmic thiredoxin	CO	-0.64
Cj1665		Possible lipoprotein thiredoxin	CO	-24.24
Cj1666c		CopG protein	S	1.69
Cj1669c		DNA ligase (ATP) (EC 6.5.1.1)	L	24.24
Cj1670c	cgpA	Putative periplasmic protein	S	-2.32
Cj1671c		hypothetical protein	-	-2.01
Cj1672c	eno	Enolase (EC 4.2.1.11)	G	-0.94
Cj1673c	recA	RecA protein	L	-1.46
Cj1674		1,4-dihydroxy-6-naphtoate synthase	H	2.31
Cj1676	murB	UDP-N-acetylenolpyruvoylglucosamine reductase (EC 1.3.1.98)	M	-25.06
Cj1677		hypothetical protein	U	0.08
Cj1679		hypothetical protein	M	-0.73
Cj1681c	cysQ	3'(2'),5'-bisphosphate nucleotidase (EC 3.1.3.7)	P	0.44
Cj1682c	gltA	Citrate synthase (si) (EC 2.3.3.1)	H	-1.06
Cj1684c		NA+/H+ antiporter (napA), putative	P	-0.55
Cj1685c	bioB	Biotin synthase (EC 2.8.1.6)	H	-0.97
Cj1686c	topA	DNA topoisomerase I (EC 5.99.1.2)	L	1.10
Cj1688c	secY	Protein translocase subunit SecY	U	-26.61
Cj1689c	rplO	LSU ribosomal protein L15p (L27Ae)	J	25.17
Cj1690c	rpsE	SSU ribosomal protein S5p (S2e)	J	-0.77
Cj1691c	rplR	LSU ribosomal protein L18p (L5e)	J	-0.29
Cj1692c	rplF	LSU ribosomal protein L6p (L9e)	J	-0.85
Cj1693c	rpsH	SSU ribosomal protein S8p (S15Ae)	J	-1.05
Cj1695c	rplE	LSU ribosomal protein L5p (L11e)	J	-0.82
Cj1696c	rplX	LSU ribosomal protein L24p (L26e)	J	-0.55
Cj1697c	rplN	LSU ribosomal protein L14p (L23e)	J	-1.95
Cj1698c	rpsQ	SSU ribosomal protein S17p (S11e)	J	-0.65
Cj1699c	rpmC	LSU ribosomal protein L29p (L35e)	J	1.35
Cj1700c	rplP	LSU ribosomal protein L16p (L10e)	J	1.45
Cj1701c	rpsC	SSU ribosomal protein S3p (S3e)	J	-0.64
Cj1702c	rplV	LSU ribosomal protein L22p (L17e)	J	0.91

Cj1704c	rplB	LSU ribosomal protein L2p (L8e)	J	-0.98
Cj1705c	rplW	LSU ribosomal protein L23p (L23Ae)	J	-2.18
Cj1706c	rplD	LSU ribosomal protein L4p (L1e)	J	-0.43
Cj1707c	rplC	LSU ribosomal protein L3p (L3e)	J	0.47
Cj1709c		LSU rRNA pseudouridine(2605) synthase (EC 5.4.99.22)	J	0.00
Cj1710c		Ribonuclease J (endonuclease and 5' exonuclease)	J	2.06
Cj1711c	ksgA	SSU rRNA (adenine(1518)-N(6)/adenine(1519)-N(6))-dimethyltransferase (EC 2.1.1.182)	J	0.00
Cj1712		purine nucleoside phosphorylase (punB)	F	24.98
Cj1713		23S rRNA (adenine(2503)-C(2))-methyltransferase @ tRNA (adenine(37)-C(2))-methyltransferase (EC 2.1.1.192)	J	5.17
Cj1715		Acetyltransferase	K	1.11
Cj1716c	leuD	3-isopropylmalate dehydratase small subunit (EC 4.2.1.33)	E	3.53
Cj1717c	leuC	3-isopropylmalate dehydratase large subunit (EC 4.2.1.33)	E	3.17
Cj1718c	leuB	3-isopropylmalate dehydrogenase (EC 1.1.1.85)	CE	0.40
Cj1719c	leuA	2-isopropylmalate synthase (EC 2.3.3.13)	E	-0.30
Cj1720		hypothetical protein	-	1.92
Cj1724c		NADPH-dependent 7-cyano-7-deazaguanine reductase (EC 1.7.1.13)	S	-24.18
Cj1725		Putative periplasmic protein	O	-1.06
Cj1726c	metA	Homoserine O-succinyltransferase (EC 2.3.1.46)	E	-0.79
Cj1727c	metB	O-acetylhomoserine sulfhydrylase (EC 2.5.1.49) @ O-succinylhomoserine sulfhydrylase (EC 2.5.1.48)	E	-0.01
Cj1729c	flgE	Paralog of flagellar hook protein FlgE	N	-0.62
Cj1731c	ruvC	Crossover junction endodeoxyribonuclease RuvC (EC 3.1.22.4)	L	28.04

Table. S2A. KEGG pathways of upregulated proteins from *C. jejuni* strain NCTC 11168 biofilms.

General description	Functional category	KEGG pathway	Count
	Global and overview maps	01100 Metabolic pathways	49
		01110 Biosynthesis of secondary metabolites	22
		01120 Microbial metabolism in diverse environments	7
		01200 Carbon metabolism	3
		01210 2-Oxocarboxylic acid metabolism	4
		01212 Fatty acid metabolism	2
		01230 Biosynthesis of amino acids	10
		01232 Nucleotide metabolism	2
		01250 Biosynthesis of nucleotide sugars	6
		01240 Biosynthesis of cofactors	17
		01220 Degradation of aromatic compounds	0
	Carbohydrate metabolism	00010 Glycolysis / Gluconeogenesis	1
		00020 Citrate cycle (TCA cycle)	0
		00030 Pentose phosphate pathway	0
		00040 Pentose and glucuronate interconversions	0
		00051 Fructose and mannose metabolism	0
		00052 Galactose metabolism	1
		00500 Starch and sucrose metabolism	0
		00520 Amino sugar and nucleotide sugar metabolism	4
		00620 Pyruvate metabolism	1
		00630 Glyoxylate and dicarboxylate metabolism	1
		00640 Propanoate metabolism	1
		00650 Butanoate metabolism	0
		00660 C5-Branched dibasic acid metabolism	2
		00562 Inositol phosphate metabolism	0
	Energy metabolism	00190 Oxidative phosphorylation	2
		00680 Methane metabolism	1
		00910 Nitrogen metabolism	1
		00920 Sulfur metabolism	0
	Lipid metabolism	00061 Fatty acid biosynthesis	2
		00121 Secondary bile acid biosynthesis	0
		00561 Glycerolipid metabolism	1
		00564 Glycerophospholipid metabolism	2
		00565 Ether lipid metabolism	1
		00592 alpha-Linolenic acid metabolism	1
	Nucleotide metabolism	00230 Purine metabolism	1
		00240 Pyrimidine metabolism	2
**Metabolism**	Amino acid metabolism	00250 Alanine, aspartate and glutamate metabolism	0
		00260 Glycine, serine and threonine metabolism	3
		00270 Cysteine and methionine metabolism	1
		00280 Valine, leucine and isoleucine degradation	0
		00290 Valine, leucine and isoleucine biosynthesis	3
		00300 Lysine biosynthesis	1
		00220 Arginine biosynthesis	1
		00330 Arginine and proline metabolism	1
		00340 Histidine metabolism	2

		00350	Tyrosine metabolism	0
		00360	Phenylalanine metabolism	0
		00380	Tryptophan metabolism	0
		00400	Phenylalanine, tyrosine and tryptophan biosynthesis	1
	Metabolism of other amino acids	00410	beta-Alanine metabolism	0
		00430	Taurine and hypotaurine metabolism	0
		00450	Selenocompound metabolism	0
		00460	Cyanoamino acid metabolism	0
		00470	D-Amino acid metabolism	0
		00480	Glutathione metabolism	0
	Glycan biosynthesis and metabolism	00540	Lipopolysaccharide biosynthesis	5
		00541	O-Antigen nucleotide sugar biosynthesis	3
		00550	Peptidoglycan biosynthesis	0
		00552	Teichoic acid biosynthesis	0
		00543	Exopolysaccharide biosynthesis	0
	Metabolism of cofactors and vitamins	00730	Thiamine metabolism	2
		00740	Riboflavin metabolism	1
		00750	Vitamin B6 metabolism	0
		00760	Nicotinate and nicotinamide metabolism	1
		00770	Pantothenate and CoA biosynthesis	1
		00780	Biotin metabolism	4
		00790	Folate biosynthesis	3
		00670	One carbon pool by folate	2
		00860	Porphyrin metabolism	2
		00130	Ubiquinone and other terpenoid-quinone biosynthes	4
	Metabolism of terpenoids and polyketides	00900	Terpenoid backbone biosynthesis	1
	Biosynthesis of other secondary metabolites	00332	Carbapenem biosynthesis	0
		00261	Monobactam biosynthesis	0
		00521	Streptomycin biosynthesis	0
		00401	Novobiocin biosynthesis	0
		00999	Biosynthesis of various plant secondary metabolites	0
	Xenobiotics biodegradation and metabolism	00627	Aminobenzoate degradation	0
**Genetic Information Processing**	Transcription	03020	RNA polymerase	0
	Translation	03010	Ribosome	10
		00970	Aminoacyl-tRNA biosynthesis	0
	Folding, sorting and degradation	03060	Protein export	1
		04122	Sulfur relay system	0
		03018	RNA degradation	2
	Replication and repair	03030	DNA replication	3
		03410	Base excision repair	3
		03420	Nucleotide excision repair	2
		03430	Mismatch repair	4
		03440	Homologous recombination	2
**Environmental Information Processing**	Membrane transport	02010	ABC transporters	6
		03070	Bacterial secretion system	1
	Signal transduction	02020	Two-component system	8

**Cellular Processes**	Cellular community - prokaryotes	02024	Quorum sensing	2
	Cell motility	02030	Bacterial chemotaxis	11
		02040	Flagellar assembly	9
**Human Diseases**	Drug resistance: antimicrobial	01501	beta-Lactam resistance	0
		01502	Vancomycin resistance	0
		01503	Cationic antimicrobial peptide (CAMP) resistance	0

Table. S2B. KEGG pathways of downregulated proteins from *C. jejuni* strain NCTC 11168 biofilms.

General description	Functional category	KEGG pathway		Count
		01100	Metabolic pathways	204
		01110	Biosynthesis of secondary metabolites	95
		01120	Microbial metabolism in diverse environments	47
		01200	Carbon metabolism	27
		01210	2-Oxocarboxylic acid metabolism	11
	**Global and overview maps**	01212	Fatty acid metabolism	7
		01230	Biosynthesis of amino acids	41
		01232	Nucleotide metabolism	11
		01250	Biosynthesis of nucleotide sugars	16
		01240	Biosynthesis of cofactors	48
		01220	Degradation of aromatic compounds	3
		00010	Glycolysis / Gluconeogenesis	10
		00020	Citrate cycle (TCA cycle)	11
		00030	Pentose phosphate pathway	7
		00040	Pentose and glucuronate interconversions	2
		00051	Fructose and mannose metabolism	4
		00052	Galactose metabolism	1
		00500	Starch and sucrose metabolism	0
	**Carbohydrate metabolism**	00520	Amino sugar and nucleotide sugar metabolism	10
		00620	Pyruvate metabolism	12
		00630	Glyoxylate and dicarboxylate metabolism	5
		00640	Propanoate metabolism	6
		00650	Butanoate metabolism	5
		00660	C5-Branched dibasic acid metabolism	2
		00562	Inositol phosphate metabolism	2
		00190	Oxidative phosphorylation	23
	**Energy metabolism**	00680	Methane metabolism	6
		00910	Nitrogen metabolism	6
		00920	Sulfur metabolism	1
		00061	Fatty acid biosynthesis	7
		00121	Secondary bile acid biosynthesis	1
	**Lipid metabolism**	00561	Glycerolipid metabolism	2
		00564	Glycerophospholipid metabolism	4
		00565	Ether lipid metabolism	0
		00592	alpha-Linolenic acid metabolism	0
	**Nucleotide metabolism**	00230	Purine metabolism	18
		00240	Pyrimidine metabolism	10
		00250	Alanine, aspartate and glutamate metabolism	12
		00260	Glycine, serine and threonine metabolism	5
**Metabolism**		00270	Cysteine and methionine metabolism	6
		00280	Valine, leucine and isoleucine degradation	1
		00290	Valine, leucine and isoleucine biosynthesis	3
		00300	Lysine biosynthesis	6
	**Amino acid metabolism**	00220	Arginine biosynthesis	6
		00330	Arginine and proline metabolism	4
		00340	Histidine metabolism	9
		00350	Tyrosine metabolism	3
		00360	Phenylalanine metabolism	3

		00380	Tryptophan metabolism	0
		00400	Phenylalanine, tyrosine and tryptophan biosynthesis	9
	**Metabolism of other amino acids**	00410	beta-Alanine metabolism	2
		00430	Taurine and hypotaurine metabolism	0
		00450	Selenocompound metabolism	4
		00460	Cyanoamino acid metabolism	2
		00470	D-Amino acid metabolism	4
		00480	Glutathione metabolism	2
	**Glycan biosynthesis and metabolism**	00540	Lipopolysaccharide biosynthesis	11
		00541	O-Antigen nucleotide sugar biosynthesis	7
		00550	Peptidoglycan biosynthesis	12
		00552	Teichoic acid biosynthesis	0
		00543	Exopolysaccharide biosynthesis	0
	**Metabolism of cofactors and vitamins**	00730	Thiamine metabolism	6
		00740	Riboflavin metabolism	5
		00750	Vitamin B6 metabolism	4
		00760	Nicotinate and nicotinamide metabolism	2
		00770	Pantothenate and CoA biosynthesis	7
		00780	Biotin metabolism	4
		00790	Folate biosynthesis	11
		00670	One carbon pool by folate	3
		00860	Porphyrin metabolism	4
		00130	Ubiquinone and other terpenoid-quinone biosynthesi	2
	**Metabolism of terpenoids and polyketides**	00900	Terpenoid backbone biosynthesis	5
	**Biosynthesis of other secondary metabolites**	00332	Carbapenem biosynthesis	1
		00261	Monobactam biosynthesis	2
		00521	Streptomycin biosynthesis	1
		00401	Novobiocin biosynthesis	3
		00999	Biosynthesis of various plant secondary metabolites	2
	**Xenobiotics biodegradation and metabolism**	00627	Aminobenzoate degradation	1
**Genetic Information Processing**	**Transcription**	03020	RNA polymerase	1
	**Translation**	03010	Ribosome	14
		00970	Aminoacyl-tRNA biosynthesis	13
	**Folding, sorting and degradation**	03060	Protein export	9
		04122	Sulfur relay system	5
		03018	RNA degradation	5
	**Replication and repair**	03030	DNA replication	5
		03410	Base excision repair	3
		03420	Nucleotide excision repair	4
		03430	Mismatch repair	7
		03440	Homologous recombination	9
**Environmental Information Processing**	**Membrane transport**	02010	ABC transporters	20
		03070	Bacterial secretion system	9
	**Signal transduction**	02020	Two-component system	27
**Cellular Processes**	**Cellular community - prokaryotes**	02024	Quorum sensing	10
	**Cell motility**	02030	Bacterial chemotaxis	15
		02040	Flagellar assembly	15

**Human Diseases**	**Drug resistance: antimicrobial**	01501	beta-Lactam resistance	3
		01502	Vancomycin resistance	3
		01503	Cationic antimicrobial peptide (CAMP) resistance	2
